# Supplementary material for: Deciphering quantum fingerprints in electric conductance
Source: Nat Commun. 2022 Jun 8;13:3160. doi: 10.1038/s41467-022-30767-w (PMC9177777; doi:10.1038/s41467-022-30767-w)
Supplement: Supplementary file 1 — Supplementary Information [file 41467_2022_30767_MOESM1_ESM.pdf]

nature portfolio

Peer Review File

Deciphering quantum fingerprints in electric conductance

Open Access This file is licensed under a Creative Commons Attribution 4.0 International License, which permits use, sharing, adaptation, distribution and reproduction in any medium or format, as long as you give appropriate credit to the original author(s) and the source, provide a link to the Creative Commons license, and indicate if changes were made. In the cases where the authors are anonymous, such as is the case for the reports of anonymous peer reviewers, author attribution should be to 'Anonymous Referee' followed by a clear attribution to the source work. The images or other third party material in this file are included in the article's Creative Commons license, unless indicated otherwise in a credit line to the material. If material is not included in the article's Creative Commons license and your intended use is not permitted by statutory regulation or exceeds the permitted use, you will need to obtain permission directly from the copyright holder. To view a copy of this license, visit http://creativecommons.org/licenses/by/4.0/.

REVIEWER COMMENTS

Reviewer #1 (Remarks to the Author):

The authors devised a Y-shaped neural network to extract information from fingerprint patterns in magneto-conductance in a disordered quasi-1D nanowire. The trained neural network can transcribe the magnetic fingerprints into a real image of zero-field electron probability density with an impressive fidelity.

The applications of machine learning methods to problems in quantum transport are very interesting and important.

The relation between irregular magnetic fingerprints and quantum interference revealed by the neural network will improve the knowledge on quantum transport and is potentially useful. The methodology sounds reasonable.

I agree with the publication of the manuscript provided the following comments are clarified.

(1) The work needs to be put in the proper context, both from physics viewpoint and methodological viewpoint. The features of magnetic fingerprints in literature are not summarized in the introduction. It is also necessary to mention other works implementing machine learning methods in quantum transport, such as Phys. Rev. B 89, 235411 (2014); J. Phys. Chem. B 123, 2801 (2019); Phys. Rev. B 102, 064205 (2020). It is better to claim potential advantages/disadvantages of the proposed method.

(2) The generalization ability of the neural network needs more discussions. The Fermi energy (which together with details of leads, antidot potential, magnetic-field region are not given in the methods) and disorder strength are two key parameters for the proposed tight-binding model. Does the high fidelity persist under higher Fermi energy or stronger disorder?

(3) To my view, the thickness structure in Fig. 4(c) is actually not obvious. Is there exist a physical (or mathematical) quantity to characterize the difference between Fig. 4(c) and 4(d)?

(4) From Fig. 4(h), the absolute difference between (x,y) data and (x+dx, y) data is small for dx=1,2. What is the position resolution of the proposed method?

(5) For a high fidelity, the authors found that the minimum dimension of latent space is 7. I wonder whether the 7 mysterious features can be deciphered.

Reviewer #2 (Remarks to the Author):

Report on the manuscript by Dr Daimon and co-workers entitled "Deciphering Quantum Fingerprints in Electric Conductance" NCOMMS-20-50538

The manuscript is part of a growing body of literature that aims to use tools of machine and deep learning to extract information and understanding from physical systems. As such, the method has had some exciting early sucesses in fairly simple settings, but is now starting to be applied to more serious situations. As such, one must always check wether some of the more spectecular claims are indeed validated by the data and results presented. I am starting the review with this disclaimer as a general principle, but also becuase I think that the issues raised are also pertinent to the present manuscript.

As a start, let us recall what the paper does: for a given simple two-dimensional (2D) quantum systems, i.e. electrons in a slightly disordered 2D box in the presence of 2 so-called antidots, standard quantum mechanics methods are used to compute the conductance as a function of an external magnetic flux, i.e. a parametrization of an Aharonov-Bohm flux piercing the 2D box perpendicular. A quantum wave function (WF), i.e. a distribution of squared wave function intensities inside the box is then computed for, presumably, the same sample, but at zero magnetic field (zero flux). The sizes and parameters of the simulation are modest and neither the physics nor the simulations up to this point are for themselves publishable.

The novel ingredient of the manuscript is now a variational autoencoder (VAE) based training of conductance traces (called "quantum fingerprints" in the manuscript) together with the WF for each sample of anti-dot arrangement. Based on that training, it is then shown that for samples in the validation set, using solely the conductance traces as input, the VAE constructs as output a synthetic WF picture without ever doing the quantum mechanical calculation. Taken at face value, this is surprising.

However, a number of controls are missing:

[1] Obviously, the calculations of the WF images do *not* calculate wave functions at all. Rather, only wave function intensities are being calculated, i.e. wave function amplitudes without their phases. This is important since otherwise the authors whould somehow claim to have constructued

a quantum mechanical solution without ever solving a Schroedinger equations - this holy grail of quantum mechanics, i.e. the collapse of the wave functon, is surely not what the authors had in mind. Hence it is mandatory that the language is corrected throughout the manuscript, "WF" should not be used, WI for "wave function intensities" or some such abbreviation is better.

[2] What really is happening is that the VAE acts like a really smart librarian who, from just the knowledge of the conductance patterns, say the favorite books of a customer, can predict what type of book said customer might like to read. This is of course possible and does not "kill Schroedinger's cat".

[2a] It is then important to see how good the prediction WI pattern fits the actual truth. However, the reader is only offered in a single panel in Fig. 3d a clear plot of the difference between truth and prediction for a given configuration of antidots. This figure (3d) is hard to read. Instead, I would like to have seen a quantitative comparison, for example based on the mean-squared loss or, more appropriate in image analysis, an averaged cross-correlation function. Without such quantitative analysis, the results are largely meaningless: Fig. 3g and 3g only ask the reader to compare sample pictures themselves which humans are known to be pretty bad at, while in Fig. 4 e, f, g only difference between changing figures are highlighted.

[2b] It should also be checked whether the predictive power of the VAE is better than a simple correletion of the conductance traces themselve. One can easily contruct, given an unknown conductance traces (say from the validation/test set), a quantitative comparison with the existing conductance traces in the training set. I would speculate that finding the closest conductance trace (and hence also its WI picture) would have the same accuracy as what the VAE can produce.

Saying it differently, when two cunductance traces are similar, so should be their WI images. Hence instead of using the VAE, one can simply compare the conductance traces. And then construct a new WI depending on a weighted average of the closest approximate WI.

The main benefit of the VAE would then not be its construction of similar WI images, but possible its computational speed: instead of going through all traces in the dataset and computing a neighboring measure for every new trace, one can take the trained VAE and compute the new WI faster.

[3] I do not understand the significance of the "geometry images without WFs" and the thickness is Fig. 4 a+c, b+d. Simply claining that the "dual-layered structure, is due to the quantum interference of WFs" is certainly not correct. I would argue that simply the additional details in Fig. 4a gives rise to

the observed thickness, but here any such fine detail can lead to the observed thickness. The implied connection with quantum mechanics seems wishful at best.

[4] I find the paper interesting, but I also feel that it overstates its significance and interprets its results in its own favour. In particular, it is well-known that trial/test wave functions are useful to study, say the simple product/Jastrow type or the Laughlin wave function. But what Hamiltonian actually belong to said wave function is an unsolved inverse problem. Here, the authors indirectly say that their constructed wave function images correspond to a Hamiltonian formed by two anti-dots at different positions. This is in effect the same claim. But even for simple analytic wave functions, we do not know how to reconstruct the Hamiltonian, hence I a, very doubtful about the claims shown here - even forgetting the WF vs WI point raised above.

A few minor issues:

[a] the abtract talks about how " the interference pattern ... is too complicated for humanity to understand". This is not true, even by the authors own remarks, i.e. we can understand many interference patterns quite well (Young etc). Hence the statement, in its sweeping breath, should be de-"sensationalized".

[b] The size of the nanowire is indicated at 60*50=3000 sites, but the WF images are 3600 pixels. What is happening here? In the methods section the sums go to 60 for both x and y, so is there a typo?

[c] Why is the choice of the latent space to be 7-dimensional? Have other dimensions been tried and are they all worse, by how much? Is there any understanding why "7" seems to be optimum, i.e. are there any ideas as to why this might correspond to a good representation of the essential degrees of freedom/information needed? A better analysis of latent space should be given.

[d] RMS is not enough for image comparison as it only produces an average measure of comparison which is know to fail for details (e.g. eyes in cats in simple ML/DL applications). Better is to plot differences as in Fig. 3d. In Fig. 3 of the supplement, the scale is too weak to allow proper scrutiny. Also, it would be necessary to compare/correlate with positions of the anti-dots. I would expect that around the anti-dots, the deviations are largest.

[e] The architecture of the Conv2D layers seems arbitrary and not connected to the figure resolution. A discussion as to why the stated number of layers and feature maps was chosen would be good.

In concluson, this is an interestng paper and topic. But it overstates its conclusions and does not yet fully perform all checks that are needed before publication.

Reviewer #3 (Remarks to the Author):

!--

!General report

!--

The manuscript "Deciphering Quantum Fingerprints in Electric Conductance" deals with the use of machine learning approaches to perform the inverse quantum problem of extracting an image of the electronic wave function distribution of a nanostructure from its magnetoconductance characteristics.

This enables to get an information about the initial distribution of defects, and disorder in the nanostructure from the knowledge of its magnetoconductance characteristics.

The topic and the methods of this theoretical paper are interesting, relevant and certainly in time with respect to the quantum transport community (both for theoreticians and experimentalists).

The results sound correct as far as I can see.

However, I regret the following drawbacks :

i) the writing of the paper is to me quite unclear, with many vague or overselling terms. Some efforts should be made to improve the writing with more precise and appropriate scientific language, and clearer descriptions of the theoretical model and its limitations.

The lack of clarity in some paragraphs and sentences is to me quite annoying, and induce a real difficulty for the reader to understand the paper.

Many Figs. in the paper lack proper labeling of axes.

ii) the references are to me incomplete and miss several recent theoretical advances (2019-2020) in the literature investigating quantum transport in disordered nanostructures, graphene, DNA, etc.. in which machine learning approaches are used and quantum inverse problems applied to disordered systems.

The authors should state in which sense their approach is new compared to those refs.

It is indeed difficult to evaluate the novelty of the authors' approach : they use known available software for computing conductance, and maybe known machine learning algorithms ?

So here an effort should be made in emphasizing the novelty.

Also, the difficulty in such calculations is the dependence with the system size.

Nothing is said about that in the paper (is the algorithm faster than others existing in the literature) and the corresponding limitations for the algorithm efficiency ?

iii) the magnetoconductance fingerprints discussed by the authors dependent also on energy (gate voltage) and not only on B-field.

I suspect that this energy dependence of the magnetoconductance leads to different magnetoconductance fingerprints, and thus additional information that is not deciphered by the authors' algorithm.

Also the fingerprints should get more complex upon increasing the number of antidots defects. How is the authors algorithm behaving upon increasing the number of antidots (why only 2 antidots) ? Is the algorithm able to deal several antidots and perform the inverse problem ?

iv) several checks/tests of the calculations are missing and should be addressed for asserting about the validity of the numerical procedure.

I suggest for instance checking the convergence of the disorder averaged conductance <G> upon increasing the number of disorder configurations (only 5 used in the paper and <G> is necessary to extract the fluctuations \Delta G of a given configuration),

and checking the magnetoconductance with respect to some symmetry transformations (symmetry planes, B -> - B behavior).

For those reasons, I cannot accept this paper for publication in Nature Communication in the present state.

I provide in the following detailed comments and suggestions that I hope to be useful and constructive in order to improve the writing of the paper.

!--

!Scientific report

!--

!--

I. Abstract and introduction.

I.1. About the sentences : "Although the interference pattern carries such microscopic information, it is too complicated for humanity to understand or make use of.", "The present result augments the human ability to identify quantum states, and it should allow nondestructive microscopy of quantum nanostructures in materials by making use of quantum fingerprints."

I am not convinced by those sentence, because of the use of the vocabulary "too complicated for humanity to understand", "The present result augments the human ability" refer to global

"human" general skills or goals, that I find too general, overselling, and not appropriate to scientific language which is obviously more focused and humble.

Indeed, I can admit in the title the use of the word Deciphering" as an analogy for making the paper more striking, but it seems that the authors take the analogy maybe too seriously.

For instance, what is the meaning of the word "understand" when applied to universal conductance fluctuations. Philosophically speaking to me, there is no hidden "meaning", or "message" that would need to be "unraveled" in the magnetoconductance oscillations. There is rather a fingerprint of the

disorder configuration for a given nanocircuit, the conductance of which is measured. The authors main claim seems to be that machine learning enables to "invert" this fingerprint pattern to propose an image of the nanostructure disorder configuration (encoded into the "electronic wave functions").

Regarding the comment about "nondestructive microscopy of quantum nanostructures", I do not understand the word "nondestructive" that is ambiguous. Is there a connection with non-destructure quantum measurements (which I would not understand...) ?

Also I can see additional restrictions of this "understanding" claimed by the authors, due to the fact that there model used as input of the machine learning process, does not take into account crucial physical mechanism in mesoscopic experiments, like inelastic scattering (by phonons, electrons) experimentally at the origin of a finite electronic phase coherence length (that might be larger than the elastic mean free path but still finite), and finite temperature.

I.2. "These complex patterns of electric conductance are called conductance fluctuations or quantum fingerprints in electric conductance. The fingerprint thus carries information concerning the quantum electron states."

First of all, the amplitude of the mesoscopic conductance fluctuation should be universal. Is it always the case in their calculation ?

Regarding the fingerprints that should provide information about "the quantum electron states" : I would rather think that they contain information about the elastic scattering process of electrons on the sample defects. Am I also correct to think that more precisely, the output of the machine learning calculation contains information about the square modulus of the electronic scattering states (what the author mean precisely by the electronic wave functions)? Can the authors access to real and imaginary par to their "wave-functions" and thus get information about scattering phases (important in topological materials) ?

I.3. "Nevertheless, it has been impossible to interpret it due to its extreme complexity". What do the author mean by "impossible to interpret" ? What is impossible ?

I.4. Fig.4a is commented on line 54 for the first time, so before Fig.3 is commented (the former appearing for the first time on line 86). This is a bit annoying for the reader.

!---

II. Results : Calculation of wave functions and conductance

II.1. I do find that the description of the theoretical method and modelling of the system is unclear and badly explained in the text.

For instance, no information is provided about how many electron per sites (and thus atomic wave function used for the tight-binding model), the geometry or connectivity of the lattice, how to include magnetic field (Peirls phase in which gauge ?) in the tight-binding model, what is really computed (2 point or 4 point conductance ? Connection to Landauer formula ? Which bias and gate voltage is used for the conductance calculation ?). Some information is written later in Methods part, but in this results section, the writing should be more precise.

II.2. About the sentences "For simplicity, defects in the nanowire are introduced as two antidots with a radius of 5 sites.", and "With 1591 different antidot positions and 5 different random potentials applied to the systems, 7955 samples are prepared as a dataset".

It is quite unclear in the writing of this section how disorder is introduced in the model : on-site Anderson disorder ? Which amplitude compared to hopping strength ? Why only "5 different random potentials", while the size of the set of possible disorder configurations is much larger ? What is the role of the disorder strength upon increasing it with respect to either the hopping parameter, or the antidots onsite energy ?

How are the 2 antidots defects modeled in the tight-binding model. Why only 2 antidots and not more ? Is there a reason for this (limitation in the size of the system for the calculation or difficulties in "interpreating" the WF 2D map if more than 2 antidot defects ?) In other words how robust is the algorithm of the authors upon increasing the number of antidots ?

II.3. "we introduce the normalised magneto-conductance ΔG(ϕ) calculated by subtracting the averaged G over all the dataset from the raw G(ϕ) data".

*The conductance <G> averaged over disorder configurations is substracted from the conductance G obtain in a particular sample.

Is this averaged conductance converged with only 5 disorder configurations ?

*The authors computed only the dependence of \Delta G with the magnetic flux \phi. But I also suspect a dependence with the gate voltage (Fermi level position in the nanowire) for instance.

I suspect this energy dependence of the magnetoconductance to lead to different magnetoconductance fingerprints, and thus additional information that is not deciphered by the authors' algorithm.

Can the authors comment about this point ?

!--

III. Results : Quantum geometric decoder network

III.1. Same lack of clarity in the explanations as the previous section.

Description of Figs are not sufficient.

III.2. About the sentences : "The network is trained so that the output image matches the input image" and "the latent space of the QGD acquires minimum essential information to reconstruct the WF image". What is the input, what is the output image (the reader has to find this information on the Fig. but absent from the text) ? What is the "minimum essential information" ?

Is this concept uniquely defined ?

!--

IV. Results : Discussion

IV.1. "This demonstrates the excellent compressing ability of the feature extraction network. The above decipherment using the QGD means that the present network can understand the quantum fingerprints in magneto-conductance."

Again I am not convinced by the use of the word "understand" when stating about the compressing ability of the feature extraction network.

IV.2. "In the network, information on the interference is compressed into the 7-dimensional latent space". Why 7 dimensions for the latent space. Is this number obtained after trials and errors to reach sufficiently low RMS error between input and output ?

IV.3. "In order to understand the geometric structure, we performed a control experiment using geometry images without WFs". What does that mean "without WFs" ?

IV.4. "The result suggests that the thickness structures, such as the dual-layered structure, is due to the quantum interference of WFs." I do not understand this sentence nor the following paragraph of explanations that is to me unclear. What is the physical origin of the even-odd effect in the 2nd antidot position ? Isn't it that by shifting the position of one antidot with respect to the other, one changes the electronic interference pattern ? But I do not see in this mechanism an even-odd dependence and periodicity, unless some symmetry is involved (which one ?), or some dependence of the boundary conditions with the parity of the number of atomic sites ?

!--

V. About references

The references are to me incomplete and completely miss recent theoretical advances (2019-2020) investigating quantum transport in disordered nanostructures, graphene, DNA, using machine learning approaches and quantum inverse problems to disordered systems.

The authors should state in which sense their approach is new compared to these refs.

Indeed, as far as I can see, it is difficult to evaluate the novelty of the authors approach : they use known available software for computing conductance, and maybe known machine learning algorithms ?

!--

VI. About Figs.

VI.1. Fig.1c : The x-axis is written to be the magnetic field, while it is the magnetic-flux in units of flux quantum.

VI.2. Fig.2-b : what is the unit of the color code (what the meaning of p(site^-1) not explained in the caption?)?

VI.3. Fig.3-e : no scales, no axis for the plots : do they have all the same scale ?

VI.4. Would it be possible to generate the \Delta G(\phi) characteristics of two different antidots configurations that are mirror image one to each other with respect a plane of symmetry passing through the center of the nanowire (and through the 1st antidot, the 2nd antidot being mirror imaged in the 2 configurations) ?

How are related the two corresponding \Delta G(\phi) curves for mirror image disorder configuration ? There should be a symmetry constraint for this case, that could be also a test of the validity of the conductance calculation of the authors.

By curiosity, what are the \Delta G(\phi) upon changing B -> - B in the authors's calculations ?

[Reply to the comments of Referee #1]
We appreciate the referee for the valuable time spent on the evaluation of our manuscript and recommendation of its publication. We have carried out the additional numerical experiments proposed by the referee. We are sure that the obtained results further reinforce the discussion in the paper. We have carefully revised the manuscript in accordance with the comments. The authors' reply to the comments and revised parts of the manuscript are detailed below.

(Comment 1-1)
The work needs to be put in the proper context, both from physics viewpoint and methodological viewpoint. The features of magnetic fingerprints in literature are not summarized in the introduction. It is also necessary to mention other works implementing machine learning methods in quantum transport, such as Phys. Rev. B 89, 235411 (2014); J. Phys. Chem. B 123, 2801 (2019); Phys. Rev. B 102, 064205 (2020). It is better to claim potential advantages/disadvantages of the proposed method.

(Answer 1-1)
Thank you very much for the comments. Following the referee's comments, in the revised manuscript, we added a sentence on the previous works on the machine learning methods for quantum transport [Phys. Rev. B **89**, 235411 (2014); J. Phys. Chem. B **123**, 2801 (2019); Phys. Rev. B **102**, 064205 (2020).] and on the present method (Lines 47 – 48, Page 2).

(Comment 1-2)
The generalization ability of the neural network needs more discussions. The Fermi energy (which together with details of leads, antidot potential, magnetic-field region are not given in the methods) and disorder strength are two key parameters for the proposed tight-binding model. Does the high fidelity persist under higher Fermi energy or stronger disorder?

(Answer 1-2)
We thank the referee for bringing up the point, which was not well described in the original manuscript. Following the referee's instruction, we have carried out two controlled numerical experiments. In Supplementary Figs. 6 and 7, we show some training results of the network with different tight-binding parameters: the Fermi energy and disorder potential, respectively. The result shows that the network exhibits high fidelity regardless of the tight-binding parameters. Although the interference fringe patterns in the wave function intensity images largely depend on the Fermi energy and disorder potential (Supplementary Figs. 6 and 7), our method can reconstruct wave intensity images owing to the high flexibility of the present method.
Following the referee's comments, we added sentences on the generalization ability of the neural network to the main text (Lines 109 – 111, Page 4) and Methods (Lines 243 – 251, Page 9), and added the details of the tight-binding model to Methods (Lines 174 – 188, Page 7).

(Comment 1-3)
To my view, the thickness structure in Fig. 4(c) is actually not obvious. Is there exist a physical (or mathematical) quantity to characterize the difference between Fig. 4(c) and 4(d)?

(Answer 1-3)

Thank you very much for the comment. Following the referee's comment, we quantitatively estimated the difference between the data in Fig. 3c and d by calculating thicknesses of the data structures as the variance of the data points in the thickness direction (please note that Figs. 3 and 4 are replaced in the revised manuscript as mentioned in Answer 3-9). Firstly, as shown in Supplementary Fig. 11a, we cut out a part of the data structure in the latent space, which locally forms two-dimensional plane with a thickness. The local variance was calculated for the in-plane and thickness directions. The ratio of the local variance along the in-plane and thickness directions is 0.10 for the data structure formed by the geometry images with wave function intensities (WIs) [Supplementary Fig. 11c]. On the other hand, the data structure formed by the geometry images without WIs exhibits a much smaller value of the ratio, 0.01 [Supplementary Fig. 11b and d], showing a quantitative difference between Fig. 3c and d. To clarify this point, we added sentences to the main text (Lines 140 – 144, Pages 5 – 6) and Methods (Lines 260 – 267, Page 10).

(Comment 1-4)

From Fig. 4(h), the absolute difference between (x,y) data and (x+dx, y) data is small for dx=1,2. What is the position resolution of the proposed method?

(Answer 1-4)

Thank you very much for the comment. Following the referee's comment, we added a sentence on the point (Lines 179 – 180, Page 7). The tight-binding model we used does not fix the lattice constant and it is spatially scale-free calculation [Groth, C. *et al.*, *New J. Phys.* **16**, 063065 (2014)]. The magnitude of the absolute difference between the (x,y) data and the $(x+\mathrm{d}x,y)$ data in Fig. 3h is due to the normalization condition of the wave functions. A WI image is divided into 3600 pixels and the sum of the intensity over each image is normalized to 1 (the average pixel intensity is around 10^{-4}). Then, the magnitude of the absolute difference is around 10^{-5} as shown in Fig. 3h.

(Comment 1-5)

For a high fidelity, the authors found that the minimum dimension of latent space is 7. I wonder whether the 7 mysterious features can be deciphered.

(Answer 1-5)

We thank the referee for bringing up this point. Supplementary Fig. 5 shows the latent-space dimension dependence of the averaged RMS error of the decoded WI images. For the feature extraction network, the latent-space dimension less than 5 was found to show large RMS errors, which can be attributed to insufficient capacity of the latent space as shown in Supplementary Fig. 5a, b. On the other hand, higher latent-space dimension than 9 was found to show large RMS errors due to too many learning parameters. Due to the competition between the two, the RMS error takes a minimum around at dimension = 7. A similar behavior was also found for the WI images decoded from the geometry generative network (Supplementary Fig. 5c, d). Therefore, we determined to use the 7-dimensional latent space. To clarify the point, we added sentences to Methods (Lines 234 – 242, Page 9).

[Reply to the comments of Referee #2]
We thank the referee for the valuable time spent on the evaluation of our manuscript and positive comments. Based on the suggestions, we have carried out the additional experiments proposed by the referee, and we carefully revised the manuscript. We are sure that the obtained result further reinforces the discussion in the paper. In the following, we reply to the referee's questions and comments.

(Comment 2-1)
*Obviously, the calculations of the WF images do *not* calculate wave functions at all. Rather, only wave function intensities are being calculated, i.e. wave function amplitudes without their phases. This is important since otherwise the authors whould somehow claim to have constructued a quantum mechanical solution without ever solving a Schroedinger equations - this holy grail of quantum mechanics, i.e. the collapse of the wave functon, is surely not what the authors had in mind. Hence it is mandatory that the language is corrected throughout the manuscript, "WF" should not be used, WI for "wave function intensities" or some such abbreviation is better.*

(Answer 2-1)
Thank you very much for the comments. Following the referee's suggestion, we change "wave function (WF)" to "wave function intensity (WI)" in the revised manuscript.

(Comment 2-2a-1)
What really is happening is that the VAE acts like a really smart librarian who, from just the knowledge of the conductance patterns, say the favorite books of a customer, can predict what type of book said customer might like to read. This is of course possible and does not "kill Schroedinger's cat".

(Answer 2-2a-1)
We totally agree the point.

(Comment 2-2a-2)
It is then important to see how good the prediction WI pattern fits the actual truth. However, the reader is only offered in a single panel in Fig. 3d a clear plot of the difference between truth and prediction for a given configuration of antidots. This figure (3d) is hard to read. Instead, I would like to have seen a quantitative comparison, for example based on the mean-squared loss or, more appropriate in image analysis, an averaged cross-correlation function. Without such quantitative analysis, the results are largely meaningless: Fig. 3g and 3g only ask the reader to compare sample pictures themselves which humans are known to be pretty bad at, while in Fig. 4 e, f, g only difference between changing figures are highlighted.

(Answer 2-2a-2)
We thank the referee for bringing up the point. Following the referee's suggestion, we analyzed the WI images in terms of the normalized cross correlation:

$$R_{\mathrm{NCC}} = \frac{\sum_{i,j} A(i,j)B(i,j)}{\sqrt{\sum_{i,j} A(i,j)^2 \sum_{i,j} B(i,j)^2}},$$

where A and B are the original and deciphered WI images, respectively. As shown in Supplementary Fig. 9a, b, R_{NCC} increases with the training epoch. R_{NCC} after the training is much greater ($R_{\mathrm{NCC}} > 0.997$) than that before the training. We confirmed that the generated WI images show high fidelity in terms of the normalized cross correlation. Following the referee's comment, we added sentences to Methods (Lines 268 – 274, Page 10).

(Comment 2-2b-1)

It should also be checked whether the predictive power of the VAE is better ...

(Answer 2-2b-1)

Thank you for the question. The ultimate deciphering of the conductance fingerprint should be done just from the conductance traces themselves. In practice, on the other hand, it is not easy to learn to evaluate correlation (which can realize the generation of WI images) from the conductance data only (please see our answer 2-2c). In the present paper, we succeeded in analyzing the conductance data with the aid of the WI images. Furthermore, another application of the present method is the image generator; this method can be used for generating images of WI and the sample geometry (a kind of microscope). For the purpose, the sharpness of the output image is quite important. The sharpness of the image generation is a typical feature of the VAE. In the revised manuscript, we added a sentence on the point to the main text (Lines 116 – 118, Page 5).

(Comment 2-2b-2)

It should also be checked whether the predictive power of the VAE is better than a simple correletion of the conductance traces themselve. One can easily contruct, given an unknown conductance traces (say from the validation/test set), a quantitative comparison with the existing conductance traces in the training set. I would speculate that finding the closest conductance trace (and hence also its WI picture) would have the same accuracy as what the VAE can produce.

(Answer 2-2b-2)

Thank you for the comment. Following the referee's comment, we added a sentence to Methods (Lines 216 – 217, Page 8).

The following is the results of principal component analysis (PCA), which is a typical method to estimate correlation, on the conductance data used in the present study. In Fig. R1a, we plot the conductance traces in the 3-dimensional principal subspace of the PCA. In contrast to the result of the VAE shown in Fig. R1b (2-dimensional layer structures reflecting the antidot position), the PCA cannot well extract the antidot position (scattered and unaligned data points). Therefore, at least at this stage, PCA cannot well interpret the conductance data in terms of the defect position.

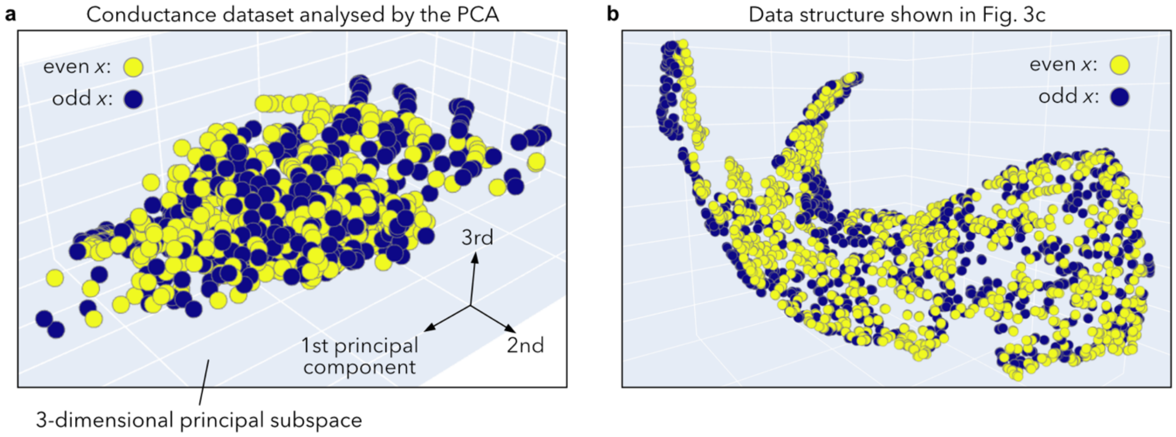

Figure R1: Principal component analysis (PCA) on the conductance traces. a, Output from the PCA analysis on the conductance plotted in the 3-dimensional principal subspace. **b**, Data structure shown in Fig. 3c (output of the feature extraction network).

(Comment 2-2c)

The main benefit of the VAE would then not be its construction of similar WI images, but possible its computational speed: instead of going through all traces in the dataset and computing a neighboring measure for every new trace, one can take the trained VAE and compute the new WI faster.

(Answer 2-2c)

Thank you for the suggestion. Please see our answer 2-2b-1. Also, we would like to add another essential point: the VAE is suitable for generating geometry images with continuously changing parameters. Owing to the variational Bayesian approach of the VAE (please refer Ref. 13 in the manuscript), similar images are placed well close together in the latent space. Therefore, we can obtain 2-dimensional curved surfaces, reflecting the antidot patterns, in the latent space. In contrast, a simple correlation analysis using the PCA cannot extract the antidot position information as shown in Fig. R1a. Therefore, the VAE makes it easier to generate a WI image from a quantum fingerprint by making the well-organized data structure in the latent space. To clarify this point, we added a sentence to the main text (Lines 131 – 132, Page 5).

(Comment 2-3a)

The implied connection with quantum mechanics seems wishful at best.

(Answer 2-3a)

Thank you for the comment. Following the referee's comment, we revised a sentence in the main text (Lines 160 – 161, Page 6).

Above all, it is not quantum mechanics that we are discussing here. We should have stated this point clearer. In fact, in the present paper, we mentioned "quantum correction" (Lines 144 – 146, Page 6 in the previous version of the manuscript), or quantum-mechanical transport, which is more specifically defined in the context of condensed matter physics.

Quantum correction in transport is defined, in condensed matter physics, as the transport properties beyond the standard Boltzmann transport (with tree-level scattering) [Lee, P. A., & Ramakrishnan, T. V. Disordered electronic systems. *Rev. Mod. Phys.* **57**, 287-337 (1985)].

One of the well-known quantum correction effects is the quantum fingerprint, which we tried to decipher in the present paper. Thanks to the referee's comment, we now realized that the word "quantum correction" needs more explanation. In the revised version, we revised a sentence on the quantum correction (Lines 160 – 161, Page 6).

(Comment 2-3b)

I do not understand the significance of the "geometry images without WFs" and the thickness is Fig. 4 a+c, b+d.
Simply claining that the "dual-layered structure, is due to the quantum interference of WFs" is certainly not correct.

(Answer 2-3b)

Thank you for the comment. Following the referee's comment, we added sentences to the main text (Lines 140 – 144, Pages 6 – 7). Please let us add some explanation.

In the discussion part, we analyze the geometric feature of the data in the latent space since geometry has the power to extract essence from complex objects in general.
The argument is simple syllogism:
A: In the latent space, data is accumulated enough to reproduce the resistance fluctuation called "quantum fingerprint" for many samples with defects. (By using the network C, the resistance fluctuation can be reproduced from the data in the latent space.)
B: The latent space data was generated via the present machine learning procedure from the WI images with the defects.
C: By comparing (i) the latent-space data generated from the wave-function interference pattern images + defect position images and (ii) the latent-space data generated from the defect position images only, in the latent space, the data-point scattering (= thickness) along the thickness direction of the data structure was found to appear only in the case (i) in all the calculation we have done.
D: A+B+C leads to the present conclusion: the strong correlation between the interference pattern in the real space and the data-point scattering along the thickness direction in the latent space.
(Classical transport should be reproduced from defect images only using Ohm's law with a resistivity parameter. On the other hand, the quantum fingerprint is a typical phenomenon attributed to the wave-function "non-local" quantum interference of conduction electrons. Please also refer to our answer 2-3a.)

Thanks to the referee's comment, we now found that the explanation was not sufficient. We added some sentences to the main text (Lines 140 – 144, Pages 5 – 6). Furthermore, in the previous version, we call the data scattering along the thickness direction simply "thickness" or "dual layer". The wording may be confusing. We revised the wording to "data-point scattering along the thickness direction" in the new text, in response to the referee's suggestion (Lines 136 – 137, Page 5).

(Comment 2-4)

I find the paper interesting, but I also feel that it overstates its significance and interprets its results in its own favour. In particular, it is well-known that trial/test wave functions are

useful to study, say the simple product/Jastrow type or the Laughlin wave function. But what Hamiltonian actually belong to said wave function is an unsolved inverse problem. Here, the authors indirectly say that their constructed wave function images correspond to a Hamiltonian formed by two anti-dots at different positions. This is in effect the same claim. But even for simple analytic wave functions, we do not know how to reconstruct the Hamiltonian, hence I a, very doubtful about the claims shown here - even forgetting the WF vs WI point raised above.

(Answer 2-4)
We thank the referee for the insightful comment. Following the referee's comment, we added sentences to Methods (Lines 229 – 233, Pages 8 – 9). Reconstructing a Hamiltonian from wave functions (inverse problem) is difficult but, in fact, it is not impossible. At least when scattering states are known for all the incident wavenumbers, potential can be completely reconstructed [Takayanagi, K., & Oishi, M., J. Math. Phys. **56**, 022101 (2015)]. In our case, although the incident wavenumbers are limited to those on the Fermi surface, the potential shape was estimated. This can be attributed to the fact that the potential shape is restricted to the antidot shape. To discuss this point, we added sentences to Methods (Lines 229 – 233, Pages 8 – 9).

(Comment 2-5)
the abtract talks about how " the interference pattern ... is too complicated for humanity to understand". This is not true, even by the authors own remarks, i.e. we can understand many interference patterns quite well (Young etc). Hence the statement, in its sweeping breath, should be de-"sensationalized".

(Answer 2-5)
Thank you for the comment. In the revised manuscript, we changed the sentence "the interference pattern ... is too complicated for humanity to understand" to a more concrete expression "it looks so random that it has not been analysed." (Lines 24 – 25, Page 1). (In Young *et al.*, conductance fluctuation was calculated in a system with controllable potential. Reconstructing microscopic structures from conductance fluctuation has been unexplored.)

(Comment 2-6)
*The size of the nanowire is indicated at 60*50=3000 sites, but the WF images are 3600 pixels. What is happening here? In the methods section the sums go to 60 for both x and y, so is there a typo?*

(Answer 2-6)
We apologize for the unclear description about the size of the WI images. It is not a typo. Although the size of the nanowire in the calculation is 60 x 50 pixels, we added zero padding with 5 sites to the left and right ends of the nanowire. To clarify this point, we added a sentence to the main text (Lines 80 – 81, Page 3).

(Comment 2-7)

Why is the choice of the latent space to be 7-dimensional? Have other dimensions been tried and are they all worse, by how much? Is there any understanding why "7" seems to be optimum, i.e. are there any ideas as to why this might correspond to a good representation of the essential degrees of freedom/information needed? A better analysis of latent space should be given.

(Answer 2-7)
We thank the referee for bringing up this point. Supplementary Fig. 5 shows the latent-space dimension dependence of the averaged RMS error of the decoded WI images. For the feature extraction network, the latent-space dimension less than 5 was found to show large RMS errors, which can be attributed to insufficient capacity of the latent space as shown in Supplementary Fig. 5a, b. On the other hand, higher latent-space dimension than 9 was found to show large RMS errors due to too many learning parameters. Due to the competition between the two, the RMS error takes a minimum around at dimension = 7. A similar behavior was also found for the WI images decoded from the geometry generative network (Supplementary Fig. 5c, d). Therefore, we determined to use the 7-dimensional latent space. In response to the referee’s comment, we added sentences to Methods (Lines 234 – 242, Page 9).

(Comment 2-8)
RMS is not enough for image comparison as it only produces an average measure of comparison which is know to fail for details (e.g. eyes in cats in simple ML/DL applications). Better is to plot differences as in Fig. 3d. In Fig. 3 of the supplement, the scale is too weak to allow proper scrutiny. Also, it would be necessary to compare/correlate with positions of the anti-dots. I would expect that around the anti-dots, the deviations are largest.

(Answer 2-8)
Thank you for the comment. Following the referee’s instruction, in the revised version, we plotted the absolute difference images with a modified scale (color) in Supplemental Fig. 3. Supplemental Fig. 10j-l show the one-dimensional position dependence of the absolute difference error between the original and deciphered geometry images. The generated antidots are almost in the same positions as that of the original images. The error takes relatively large values around the antidot position as the referee pointed out. To clarify the point, we added a sentence to the main text (Line 117, Page 5).

(Comment 2-9)
The architecture of the Conv2D layers seems arbitrary and not connected to the figure resolution. A discussion as to why the stated number of layers and feature maps was chosen would be good.

(Answer 2-9)
Thank you for the comment. Following the referee’s comment, we added a sentence to Methods (Lines 210 – 212, Page 8).
We determined the network architecture parameters based on a recipe frequently used in machine learning for image recognition and fine-tuned it.

[Reply to the comments of Referee #3]
We thank the referee for the valuable time spent on the evaluation of our manuscript and positive comments. Following the instruction, we have carried out the additional numerical experiments proposed by the referee, and carefully revised the manuscript. We are sure that the obtained result further reinforces the discussion in the paper. In the following, we reply to the referee's questions and comments.

(Comment 3-1)
The lack of clarity in some paragraphs and sentences is to me quite annoying, and induce a real difficulty for the reader to understand the paper.
Many Figs. in the paper lack proper labeling of axes.

(Answer 3-1)
Thank you for the comment. Following the comments, we carefully rephrased or removed the vague terms (e.g., Lines 24 – 25, Page 1; Line 46, Page 2; Lines 56 – 60, Pages 3 – 4; Lines 123 – 124, Page 5) and revised the descriptions on the theoretical model (Lines 174 – 195, Page 7). We also carefully revised the labels of the axes in Figs. 1-4.

(Comment 3-2a)
the references are to me incomplete and miss several recent theoretical advances (2019-2020) in the literature investigating quantum transport in disordered nanostructures, graphene, DNA, etc.. in which machine learning approaches are used and quantum inverse problems applied to disordered systems.

(Answer 3-2a)
We thank the referee for the comment. Following the referee's comment, in the revised manuscript, we added some sentences on the previous works on the machine learning methods for quantum transport [Phys. Rev. B **89**, 235411 (2014); J. Phys. Chem. B **123**, 2801 (2019); Phys. Rev. B **102**, 064205 (2020).] (Lines 47 – 48, Page 2).

(Comment 3-2b)
The authors should state in which sense their approach is new compared to those refs.
It is indeed difficult to evaluate the novelty of the authors' approach:

(Answer 3-2b)
The present paper is essentially different from these previous literatures. The previous papers predict macroscopic observable quantity: conductance, from microscopic information. On the other hand, the present paper provides a generator which outputs microscopic images: wave function intensities (WIs) and sample geometry, from macroscopic conductance. The latter is a kind of microscopy in a sense. This is a new concept of quantum transport AI. Following the referee's comment, to clarify this point, we added a sentence to the main text (Lines 47 – 48, Page 2).

(Comment 3-2c)

Also, the difficulty in such calculations is the dependence with the system size.
Nothing is said about that in the paper (is the algorithm faster than others existing in the literature) and the corresponding limitations for the algorithm efficiency ?
they use known available software for computing conductance, and maybe known machine learning algorithms ?

(Answer 3-2c)

We thank the referee for the comment. In principle, there is no limitation on performing machine learning using data with any number of antidots. Although the essence of the present machine learning is shown in this paper, we could not perform more general learning with greater system sizes at this time due to our resource limit. As we mentioned in Answer 2-2b-2 and Answer 2-2c, we compared the data structures generated from the present network and a well-known machine learning technique, principal component analysis (PCA). We found that the PCA cannot extract the antidots pattern information. To solve this problem, we have spent time to find the present learning system. The coding was based on the PyTorch platform. The generating ability of WI images is new. It is not a matter of speed. To clarify this point, we added sentences to the main text (Lines 116 – 118, Page 5; Lines 131 – 132, Page 5).

(Comment 3-3)

the magnetoconductance fingerprints discussed by the authors dependent also on energy (gate voltage) and not only on B-field.
I suspect that this energy dependence of the magnetoconductance leads to different magnetoconductance fingerprints, and thus additional information that is not deciphered by the authors' algorithm.
Also the fingerprints should get more complex upon increasing the number of antidots defects. How is the authors algorithm behaving upon increasing the number of antidots (why only 2 antidots) ? Is the algorithm able to deal several antidots and perform the inverse problem ?

(Answer 3-3)

We thank the referee for raising the point. Following the referee's instruction, we have carried out numerical experiments. In Supplementary Fig. 8, we show WI images decoded from the Fermi energy dependence of conductance. The data shows that the WI images can be decoded with high fidelity when we use the Fermi energy dependence-data instead of the magnetic field dependence. For this point, we added sentences to Methods (Lines 248 – 251, Page 9).
We also thank the referee for bringing up the point of the number of antidot defects. In principle, there is no limitation on performing machine learning using data with any number of antidots. Although the essence of the present machine learning is shown in this paper, we could not perform more general learning with greater system sizes at this time due to our resource limit.

(Comment 3-4)

several checks/tests of the calculations are missing and should be addressed for asserting about the validity of the numerical procedure.

I suggest for instance checking the convergence of the disorder averaged conductance <G> upon increasing the number of disorder configurations (only 5 used in the paper and <G> is necessary to extract the fluctuations ¥Delta G of a given configuration),

and checking the magnetoconductance with respect to some symmetry transformations (symmetry planes, B -> - B behavior).

(Answer 3-4)

We thank the referee for raising this point. Following the referee's instruction, we carried out simulations proposed by the referee. We checked the convergence of the disorder averaged conductance <*G*> upon increasing the number of disorder configurations as shown in Fig. R2. We also checked whether the relationship required by the symmetry is satisfied. As shown in Fig. R3, the same magnetic fingerprint appears with respect to the magnetic field reversal. Theoretically, electrical conduction needs to satisfy the Onsager reciprocal relation $G_{ij,mn}(B) = G_{nm,ji}(-B)$, where the pairs ij and mn represent the leads used to apply an electric current and measure a voltage, respectively. In the case of the two-terminal conductance, the reciprocal relation leads to the simple relation $G(B) = G(-B)$. Our calculation is consistent with the Onsager reciprocal relation.

We also confirmed that the conductance fingerprint patterns are the same in samples that are spatially inverted from each other, satisfying the symmetry requirement for the two-terminal conductivity. [For the calculation, we remade sample data with a symmetric external shape, since, in the sample used for the deciphering, one of the defects was fixed at an asymmetric position (see Fig. R3).]

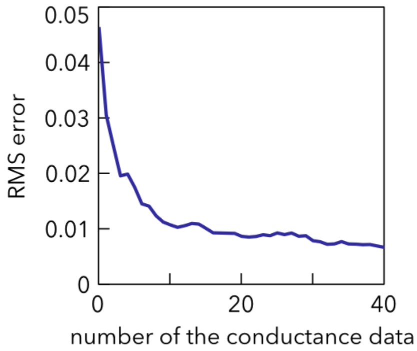

Figure R2: Convergence of the conductance data. We calculated 500 conductance traces with different disorder configurations and obtained the averaged conductance G_{ave}. To check the convergence of the averaged conductance, we plotted the RMS error:

$$\sqrt{\sum_{i=1}^{101} \left| G_{n,i} - G_{\text{ave},i} \right|^2 / 101},$$

where n is the number of the disorder configurations and G_n is the disorder averaged conductance averaged over *n* conductance traces. i is a label representing the magnetic field. The RMS error rapidly decreases with increasing the number of disorder configurations.

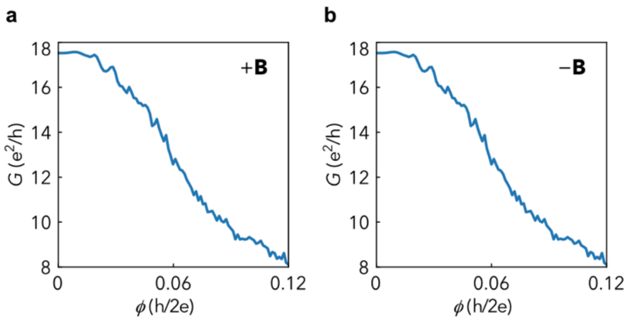

Figure R3: Comparison between the calculated magnetic fingerprints with opposite magnetic field directions

(Comment 3-5a)

About the sentences : "Although the interference pattern carries such microscopic information, it is too complicated for humanity to understand or make use of.", "The present result augments the human ability to identify quantum states, and it should allow nondestructive microscopy of quantum nanostructures in materials by making use of quantum fingerprints."

I am not convinced by those sentence, because of the use of the vocabulary "too complicated for humanity to understand", "The present result augments the human ability" refer to global "human" general skills or goals, that I find too general, overselling, and not appropriate to scientific language which is obviously more focused and humble.

Indeed, I can admit in the title the use of the word Deciphering" as an analogy for making the paper more striking, but it seems that the authors take the analogy maybe too seriously.

For instance, what is the meaning of the word "understand" when applied to universal conductance fluctuations. Philosophically speaking to me, there is no hidden "meaning", or "message" that would need to be "unraveled" in the magnetoconductance oscillations.

(Answer 3-5a)

We apologize for the confusing expression in the previous version of the manuscript. As we mentioned in Answer 3-1, we carefully rephrased or removed the vague terms (e.g., Lines 24 – 25, Page 1; Line 46, Page 2; Lines 56 – 60, Pages 3 – 4; Lines 123 – 124, Page 5), following the referee's comments.

(Comment 3-5b)

There is rather a fingerprint of the disorder configuration for a given nanocircuit, the conductance of which is measured. The authors main claim seems to be that machine learning enables to "invert" this fingerprint pattern to propose an image of the nanostructure disorder configuration (encoded into the "electronic wave functions").

(Answer 3-5b)

Thank you for the beautiful comment. We totally agree with your point. This is what we meant.

(Comment 3-5c)

Regarding the comment about "nondestructive microscopy of quantum nanostructures", I do not understand the word "nondestructive" that is ambiguous. Is there a connection with non-destructure quantum measurements (which I would not understand...) ?

Also I can see additional restrictions of this "understanding" claimed by the authors, due to the fact that there model used as input of the machine learning process, does not take into account crucial physical mechanism in mesoscopic experiments, like inelastic scattering (by phonons, electrons) experimentally at the origin of a finite electronic phase coherence length (that might be larger than the elastic mean free path but still finite), and finite temperature.

(Answer 3-5c)

We apologize for the confusion about the meaning of the word "nondestructive". We should have used the word more carefully. As pointed out by the referee, in the context of quantum mechanics, the word "nondestructive" might be reminiscent of the phenomena related to wave function collapse, such as the Schrodinger's cat problem. On the other hand, in the field of microscope engineering, "nondestructive microscope" simply means a microscope that allows a sample to be observed as it is without any processing such as scraping or drilling the surface of the sample. In this paper, we use this term to mean the latter. We apologize for the confusion. In revised version, we deleted this part (Lines 29 – 30, Page 2).

We did not take the inelastic scattering into consideration, since it is known, from an experimental point of view, that a universal conductance fluctuation pattern does not change so much below the temperature at which the electron coherence length becomes comparable to the width of the sample (e.g. around $\sim 10^{-1}$ K in submicron Au wires). Above the temperature, the amplitude of the conductance fluctuation is much less than e^2/h. Following the referee's comments, we revised a sentence on this point (Lines 71 – 73, Page 3).

(Comment 3-6)

"These complex patterns of electric conductance are called conductance fluctuations or quantum fingerprints in electric conductance. The fingerprint thus carries information concerning the quantum electron states."

First of all, the amplitude of the mesoscopic conductance fluctuation should be universal. Is it always the case in their calculation ?

(Answer 3-6)

We thank the referee for bringing up the fundamental point. We found that the fluctuation is the order of e^2/h $(\sim 0.3\, e^2/h)$, consistent with the previous UCF papers [Lee, P. A. *et al.*, Phys. Rev. B **35**, 1039 (1987)]. To mention this point, we added a sentence to the main text (Lines 193 – 194, Page 7).

(Comment 3-7)

Regarding the fingerprints that should provide information about "the quantum electron states" : I would rather think that they contain information about the elastic scattering process of electrons on the sample defects. Am I also correct to think that more precisely, the output of the machine learning calculation contains information about the square modulus of the electronic scattering states (what the author mean precisely by the electronic wave

functions)? Can the authors access to real and imaginary par to their "wave-functions" and thus get information about scattering phases (important in topological materials) ?

(Answer 3-7)
We thank the referee for commenting on the point. The output of the machine learning calculation contains information about the square modulus of the electronic scattering states. Following this comment, we replaced “wave function (WF)” with “wave function intensity (WI)” in the main text. Using real and imaginary parts of WF for the learning is an interesting idea although it is beyond the scope of this paper. We note that some of the wave function phase information is included in the amplitude because the amplitude is the results of complex interference between waves scattered by the antidots. Information about topological electronic states can also be obtained, in principle, from the wave function intensity near the edge/surface of the samples. In response to the referee’s comment, we added a sentence to Methods (Lines 195 – 196, Page 7).

(Comment 3-8)
"Nevertheless, it has been impossible to interpret it due to its extreme complexity". What do the author mean by "impossible to interpret" ? What is impossible ?

(Answer 3-8)
Following the referee’s comment, we rewrote the word to a more concrete expression (Line 46, Page 2).
From the average resistance, one can know averaged carrier parameters, such as mass and lifetime of electrons. On the other hand, the low-temperature fingerprint patterns, fluctuation patterns around the average resistance, carry real-space (thus non-local in the sense of interference) and microscopic information, such as defect positions and WIs. The direct way to make such information visible is to generate spatial images of the defects and WIs etc. from the fingerprints, which used to be difficult to carry out with a conventional way, and has yet to be done. In the revised version, we rewrote the sentence (Line 46, Page 2).

(Comment 3-9)
Fig.4a is commented on line 54 for the first time, so before Fig.3 is commented (the former appearing for the first time on line 86). This is a bit annoying for the reader.

(Answer 3-9)
Thank you very much for the comment. Following the referee’s comment, we rearranged Fig. 3 and Fig. 4 and revised the figure labels.

(Comment 3-10)
I do find that the description of the theoretical method and modelling of the system is unclear and badly explained in the text.
For instance, no information is provided about how many electron per sites (and thus atomic wave function used for the tight-binding model), the geometry or connectivity of the lattice, how to include magnetic field (Peirls phase in which gauge ?) in the tight-binding model,

what is really computed (2 point or 4 point conductance ? Connection to Landauer formula ? Which bias and gate voltage is used for the conductance calculation ?). Some information is written later in Methods part, but in this results section, the writing should be more precise.

(Answer 3-10)
Following the referee's comments, we added some detailed information on the calculation to the main text (Lines 66 – 73, Page 3) and Methods (Lines 174 – 196, Page 7). We thank the referee.

(Comment 3-11)
About the sentences "For simplicity, defects in the nanowire are introduced as two antidots with a radius of 5 sites.", and "With 1591 different antidot positions and 5 different random potentials applied to the systems, 7955 samples are prepared as a dataset".
It is quite unclear in the writing of this section how disorder is introduced in the model : on-site Anderson disorder ? Which amplitude compared to hopping strength ? Why only "5 different random potentials", while the size of the set of possible disorder configurations is much larger ? What is the role of the disorder strength upon increasing it with respect to either the hopping parameter, or the antidots onsite energy ?

(Answer 3-11)
We thank the referee for the comment. Following the referee's comments, we revised the sentences on the randomness to Methods (Lines 182 – 183, Page 7).
In this study, we introduced small disorder as on-site disorder potential, where the magnitude of the disorder potential is one tenth of the hopping energy. We prepared several disorder potentials to extract more universal information that does not depend on the details of the disorder potential (so called the data augmentation method). The number of the random potential patterns is set to 5 for each defect position. Here, we checked that 5 random potentials are enough to obtain the generalization ability to decode WI images.
To answer the referee's question that "What is the role of the disorder strength upon increasing it with respect to either the hopping parameter, or the antidots onsite energy?", we performed a control experiment, where we doubled the magnitude of the disorder potentials. Supplementally Fig. 7 shows the decoded WI images. The WI images are well reproduced even when the disorder potential amplitude is doubled. However, we found that, when the disorder potential is too large (50% of the hopping energy), the RMS error becomes quite large. It is probable that the conductance is greatly disturbed as the disorder potential energy approaches the hopping energy. Following the referee's comment, we added sentences to Methods (Lines 243 – 241, Page 9).

(Comment 3-12)
How are the 2 antidots defects modeled in the tight-binding model. Why only 2 antidots and not more ? Is there a reason for this (limitation in the size of the system for the calculation or difficulties in "interpreating" the WF 2D map if more than 2 antidot defects ?) In other words how robust is the algorithm of the authors upon increasing the number of antidots ?

(Answer 3-12)
Thank you for the comment. The antidot defect is modeled by removing lattice points in a circle shape from the 2-dimensional square lattice tight-binding sites of the nanowire. Following the referee's comment, we added sentences to Methods (Lines 186 – 188, Page 7).
As we mentioned in Answer 3-3, it is possible to perform the learning using data for any number of antidots in principle. Although the essence of the present machine learning is demonstrated in this paper on two antidots, we could not perform the learning for more general case at the moment due to our resource limit. The more general case requires much larger computational resources because the degrees of freedom of antidot patterns increase exponentially as the number of antidots increases. More generalized learning will be a future task.

(Comment 3-13)
"we introduce the normalised magneto-conductance ΔG(φ) calculated by subtracting the averaged G over all the dataset from the raw G(φ) data".
**The conductance <G> averaged over disorder configurations is substracted from the conductance G obtain in a particular sample. Is this averaged conductance converged with only 5 disorder configurations ?*

(Answer 3-13)
Thank you for the comment. We apologize for the unclear description on the averaged conductance <*G*>. We defined <*G*> as the conductance value averaged over 7955 conductance data with 1591 different antidot positions and 5 different random potentials.
We checked the convergence of the disorder averaged conductance <*G*> upon increasing the number of disorder configurations (please see Answer3-4 and Fig. R2). To clarify this point, we revised a sentence in the main text (Line 77, Page 3) and added sentences to Methods (Lines 191 – 193, Page 7).

(Comment 3-14)
**The authors computed only the dependence of ¥Delta G with the magnetic flux ¥phi. But I also suspect a dependence with the gate voltage (Fermi level position in the nanowire) for instance. I suspect this energy dependence of the magnetoconductance to lead to different magnetoconductance fingerprints, and thus additional information that is not deciphered by the authors' algorithm. Can the authors comment about this point ?*

(Answer 3-14)
We thank the referee for raising the new point. Following the referee's instruction, we have carried out additional experiments on the Fermi energy dependence of the conductance. In Supplementary Fig. 8, we show the results, showing that the neural network we proposed exhibits high fidelity even when using the Fermi-energy dependence data.

(Comment 3-15)
Same lack of clarity in the explanations as the previous section. Description of Figs are not sufficient.

(Answer 3-15)

Thank you for the comment. To clarify this point, we revised the figure panels and captions in Figs. 1-4 and Supplementary Fig. 3.

(Comment 3-16)
About the sentences : "The network is trained so that the output image matches the input image" and "the latent space of the QGD acquires minimum essential information to reconstruct the WF image". What is the input, what is the output image (the reader has to find this information on the Fig. but absent from the text) ? What is the "minimum essential information" ? Is this concept uniquely defined ?

(Answer 3-16)
We thank the referee's comment. Following the comment, in the revised version, we revised some sentences (Lines 86 – 88, Pages 3 – 4) and changed the words "minimum essential information" (Lines 91 – 92, Page 4).
The input and output are the same WI image (An autoencoder refers to a network in which input and output are the same). "Minimum essential information" is a word used in the context of autoencoders; the VAE compresses the input of the network and extracts the information necessary to reproduce the input on the output of the network. We changed the words "minimum essential information" (Lines 91 – 92, Page 4) and added some sentences (Lines 86 – 88, Pages 3 – 4).

(Comment 3-17)
"This demonstrates the excellent compressing ability of the feature extraction network. The above decipherment using the QGD means that the present network can understand the quantum fingerprints in magneto-conductance."
Again I am not convinced by the use of the word "understand" when stating about the compressing ability of the feature extraction network.

(Answer 3-17)
Following the referee's suggestion, we rewrote the word "the present network can understand ..." to "the present network can analyze the quantum fingerprints to reconstruct the microscopic images (geometry and WI) in the sample" (Lines 123 – 124, Page 5). Thank you for the comment.

(Comment 3-18)
"In the network, information on the interference is compressed into the 7-dimensional latent space". Why 7 dimensions for the latent space. Is this number obtained after trials and errors to reach sufficiently low RMS error between input and output ?

(Answer 3-18)
We thank the referee for bringing up this point. Following the referee's comment, we added sentences to Methods (Lines 234 – 242, Page 9).
Supplementary Fig. 5 shows the latent-space dimension dependence of the averaged RMS error of the decoded wave function intensity images. For the feature extraction network, the latent-space dimension less than 5 was found to show large RMS errors, which can be attributed to insufficient capacity of the latent space as shown in Supplementary Fig. 5a, b. On

the other hand, higher latent-space dimension than 9 was found to show large RMS errors due to too many learning parameters. Due to the competition between the two, the RMS error takes a minimum around at dimension = 7. A similar behavior was also found for the wave function intensity images decoded from the geometry generative network (Supplementary Fig. 5c, d). Therefore, we determined to use the 7-dimensional latent space. In response to the referee's comment, we added sentences to Methods (Lines 234 – 242, Page 9).

(Comment 3-19)

"In order to understand the geometric structure, we performed a control experiment using geometry images without WFs". What does that mean "without WFs" ?

(Answer 3-19)

Thank you for the comment. A geometry image without WFs is defined as an image with a non-zero constant value on a nanowire and zero inside the antidots and outside the nanowire. No WI images are superimposed. The constant value is determined by the normalization condition $\sum_{i=1}^{60}\sum_{j=1}^{60} X_{i,j} = 1$, where $\boldsymbol{X}$ is a image, and the suffixes i, j represent the pixel label. In response to the referee's question, we revised a sentence in the main text (Line 139, Page 5) and added sentences to Methods (Lines 200 – 203, Pages 7 – 8).

(Comment 3-20)

"The result suggests that the thickness structures, such as the dual-layered structure, is due to the quantum interference of WFs." I do not understand this sentence nor the following paragraph of explanations that is to me unclear. What is the physical origin of the even-odd effect in the 2nd antidot position ? Isn't it that by shifting the position of one antidot with respect to the other, one changes the electronic interference pattern ? But I do not see in this mechanism an even-odd dependence and periodicity, unless some symmetry is involved (which one ?), or some dependence of the boundary conditions with the parity of the number of atomic sites ?

(Answer 3-20)

Thank you for the comment. We now realized that the explanation on this point was insufficient. In response to the comment, we would like to add an explanation on it.

In the present calculation, we chose the pixel size of the interference images so that the Fermi wavelength is equivalent to 2 pixels. Under the condition, the data scattering in the latent space was found to apparently be quantized to show the most interpretable structure: the dual layers, which can be explained by the fact that a standing wave between an antidot and the sample end can be classified into two; destructive and constructive interference patterns. In fact, the dual-layer structure was found to change into a randomly scattered structure around the surface by changing the pixel size, showing that the essential feature here is the data scattering around the surface structure representing the information of the antidot positions. We call the scattering "thickness". Following the referee's comment, we revised a sentence in the main text (Line 160, Page 6) and added sentences to Methods (Lines 260 – 267, Page 10).

(Comment 3-21)
Fig.1c : The x-axis is written to be the magnetic field, while it is the magnetic-flux in units of flux quantum.

(Answer 3-21)
Following the referee's comment, we revised the unit of the *x*-axis in Fig. 1c.

(Comment 3-22)
Fig.2-b : what is the unit of the color code (what the meaning of p(site^-1) not explained in the caption?)?

(Answer 3-22)
Thank you very much for pointing this out. We revised the label of the color code in Fig. 2.

(Comment 3-23)
Fig.3-e : no scales, no axis for the plots : do they have all the same scale ?

(Answer 3-23)
Thank you very much for pointing this out. All the plots have the same scale. We added a scale bar to Fig. 4e.

(Comment 3-24)
Would it be possible to generate the ¥Delta G(¥phi) characteristics of two different antidots configurations that are mirror image one to each other with respect a plane of symmetry passing through the center of the nanowire (and through the 1st antidot, the 2nd antidot being mirror imaged in the 2 configurations) ?
How are related the two corresponding ¥Delta G(¥phi) curves for mirror image disorder configuration ? There should be a symmetry constraint for this case, that could be also a test of the validity of the conductance calculation of the authors.
By curiosity, what are the ¥Delta G(¥phi) upon changing B -> - B in the authors's calculations ?

(Answer 3-24)
Thank you very much for the interesting comment. As we mentioned in Answer 3-4, we tried the symmetry transformations following the referee's instruction. We confirmed that the conductance calculation satisfies the Onsager reciprocal relations with respect to the magnetic field reversal and the reversal of the sample shape (please see Answer 3-4).

[List of corrections]

Main text

The words “wave function (WF)” were replaced to “wave function intensity (WI)”.

The magnetic flux ϕ was converted into the magnetic flux density B.

Lines 24 – 25
(Original manuscript)
Although the interference pattern carries such microscopic information, it is too complicated for humanity to understand or make use of.

(Revised manuscript)
Although the interference pattern carries such microscopic information, it looks so random that it has not been analysed.

Lines 29 – 30
(Original manuscript)
The present result augments the human ability to identify quantum states, and it should allow nondestructive microscopy of quantum nanostructures in materials by making use of quantum fingerprints.

(Revised manuscript)
The present result augments the human ability to identify quantum states, and it should allow microscopy of quantum nanostructures in materials by making use of quantum fingerprints.

Lines 46-47
(Original manuscript)
Nevertheless, it has been impossible to interpret it due to its extreme complexity.

(Revised manuscript)
Nevertheless, it has been considered difficult to interpret it due to its extreme complexity.

Lines 47-48
(Added sentence)
In the literature (refs. 8-10), conductance was shown to be predicted from microscope information such as defect positions by using machine learning methods.

Lines 56 – 60
(Original manuscript)
By training the network to compress calculated real-space WF data onto a low dimensional latent space and to reconstruct the data from the latent-space data, the network (A) learns to compress the WF images into their minimum information in the latent space, and the network (B) to generate WF images from the compressed information (Fig. 4a).

(Revised manuscript)
By training the network to compress the calculated real-space WI data onto a low dimensional latent space and to reconstruct the data from the latent-space data, the network (A) learns to convert the WI images into a vector in the latent space so as to best reproduce the original

images using the network (B), and the network (B) to generate WI images from the compressed information represented by the vector (Fig. 3a).

Line 65
(Original manuscript)
Calculation of wave functions and conductance

(Revised manuscript)
Calculation of wave function intensities and conductance

Lines 66 – 67
(Original manuscript)
To prepare a training dataset, we perform numerical calculation; Fig. 2a shows a calculation model of the two-dimensional nanowire with a size of 60 $\times$ 50 sites.

(Revised manuscript)
To prepare a training dataset, we perform numerical calculation; Fig. 2a shows a calculation model of the two-dimensional nanowire with a size of 60 $\times$ 50 single-orbital sites.

Lines 70 – 73
(Original manuscript)
For each sample, we calculate WFs and magneto-conductance by using a tight binding method and Landauer's formula (see Methods).

(Revised manuscript)
For each sample, we calculate WIs and two-terminal magneto-conductance by using a tight-binding method and Landauer's formula (see Methods), where the magnetic field is introduced as a Peierls phase in the Landau gauge and inelastic scattering was not taken into consideration, assuming extremely low temperatures.

Lines 75 – 77
(Original manuscript)
To highlight the patterns, we introduce the normalised magneto-conductance $\Delta G(\phi)$ calculated by subtracting the averaged G over all the dataset from the raw $G(\phi)$ data (see Fig. 2d).

(Revised manuscript)
To highlight the patterns, we introduce the normalised magneto-conductance $\Delta G(B)$ calculated by subtracting the averaged G over all the 7955 conductance data from the raw $G(B)$ data (see Fig. 2d).

Lines 80 – 81
(Added sentence)
Each WI image is converted to 60 $\times$ 60 pixel data, and zero padding[15] of 5 pixels was applied to the left and right ends of the nanowire.

Lines 86 – 90
(Original manuscript)

The feature extraction neural network is based on VAE[10,11], and convolutes a WF image with 3600 pixels into a 7-dimensional latent space [(A) in Fig. 1d], and then deconvolutes it back to an image with 3600 pixels [(B) in Fig. 1d].

(Revised manuscript)
The feature extraction neural network is based on VAE[13,14]; VAE was shown to compress its input and extract the information necessary to reproduce the input on the output of the network[13]. The network converts a WI image with 3600 pixels into a 7-dimensional latent-space data [(A) in Fig. 1d], and then converts the data back to an image with 3600 pixels [(B) in Fig. 1d].

Lines 91 – 92
(Original manuscript)
After the training, the latent space of the QGD acquires minimum essential information to reconstruct the WF image.

(Revised manuscript)
After the training, the latent space of the QGD acquires essential information to reconstruct the WI images.

Lines 109-111
(Added sentence)
We also checked that similar generalisation performance of the QGD can be obtained even if some different system parameters are used (see Methods).

Lines 116 – 118
(Original manuscript)
The output image well reproduces the input WF image.

(Revised manuscript)
The output image clearly reproduces the input WI image (see Supplementary Figs. 3 and 10 for detailed error profiles), which can be attributed to the fact that the sharpness of the image generation is an advantage of the VAE.

Lines 123 – 124
(Original manuscript)
The above decipherment using the QGD means that the present network can understand the quantum fingerprints in magneto-conductance.

(Revised manuscript)
The above decipherment using the QGD means that the present network can analyse the quantum fingerprints to reconstruct the microscopic images (geometry and WIs) in the sample.

Lines 131 – 132
(Added sentence)
Owing to the variational Bayesian approach of VAE[13], VAE was found to construct well-organised data structures in the latent space.

Lines 132 – 135
(Original manuscript)

One can see that the dataset forms a 2-dimensional curved surface, but in some regions the surface shows a structure with some thickness (see, for instance, the regions with a dual-layered structure indicated by the red arrows in Fig. 4c).

(Revised manuscript)
In the present feature extraction network, one can see that the dataset forms a 2-dimensional curved surface in the latent space (Fig. 3c), but the surface shows a structure with some thickness (see, for instance, the regions with a dual-layered structure indicated by the red arrows in Fig. 3c).

Lines 136 – 137
(Original manuscript)
However, the appearance of the thickness structures cannot be explained by the location degree of freedom.

(Revised manuscript)
However, the appearance of the data-point scattering along the thickness direction cannot be explained by the location degree of freedom.

Line 139
(Original manuscript)
compare Fig. 4a, c and b, d

(Revised manuscript)
compare Fig. 3a, c and b, d and see Methods for details of the geometry images without WIs

Lines 140 – 141
(Original manuscript)
In contrast to the images with WFs, the dataset without WFs forms a simple plane without the thickness structures in the 3-dimensional representation (see Fig. 4d).

(Revised manuscript)
In contrast to the images which contain WI as well as sample-shape images, the dataset without WI images forms a simple plane without the thickness structures in the 3-dimensional representation (see Fig. 3d).

Lines 141 – 144
(Added sentences)
By comparing the data in the latent space, we found that dataset generated from the inputs with WI images is about ten times thicker than that generated from the inputs without WI images, where the thickness is defined as the variance of the data points (see Methods and Supplementary Fig. 11).

Lines 159 – 160
(Original manuscript)
… the observed dual-layered structure in the latent space.

(Revised manuscript)
… the observed dual-layered structure in the latent space (see Methods for more details).

Lines 160 – 163
(Original manuscript)

The result shows that the effect of quantum interference, a quantum correction to the conductivity, is encoded in the latent space as the thickness-dimension information, which emerges on the two-dimensional curved surface representing the classical information.

(Revised manuscript)
The result shows that the effect of quantum interference, quantum correction to the conductivity beyond the standard Boltzmann transport, is encoded in the latent space as the thickness-dimension information, which emerges on the 2-dimensional curved surface representing the classical information.

Methods

The words "wave function (WF)" were replaced to "wave function intensity (WI)".

Lines 178 – 180
(Added sentence)
$\phi_0 \equiv h/e$ is the magnetic flux quantum. The magnetic flux density B is introduced over the entire scattering region. a is the lattice constant and it is not a fixed parameter because our model is spatially scale-free calculation.

Lines 180 – 185
(Original manuscript)
The second term in the Hamiltonian is summed over the nearest-neighbor pairs, whose distance is unity. The parameters are set as $t = 1$ and $\phi = 0 \sim 0.12$ with 101 steps. U_i is sampled uniformly in the range from -0.05 to 0.05.

(Revised manuscript)
The second term in the Hamiltonian is a Peierls phase with the Landau gauge and is summed over the nearest-neighbor pairs. The hopping energy is set to $t = 1$. The magnitude of the disorder potential is set to one tenth of the hopping energy; U_i is sampled uniformly but randomly in the range from -0.05t to 0.05t. The Fermi energy is set to 2.0t except for the dataset of the Fermi-energy dependent magneto-conductance calculation in Supplementary Fig. 8.

Lines 186 – 188
(Added sentences)
The antidot is modeled by removing lattice points in a circular shape from the 2-dimensional square lattice of the nanowire. Two leads with the same square lattice and hopping parameter are attached to both ends of the scattering region.

Lines 190 – 194
(Added sentences)
B dependence of the 2-terminal conductance is calculated, where B is swept from 0 to 0.12 in 101 divisions. The normalised magneto-conductance $\Delta G(B)$ is calculated by subtracting the averaged conductance $G_{\mathrm{ave}}(B)$ from the raw $G(B)$, where we defined $G_{\mathrm{ave}}(B)$ as the conductance value averaged over 7955 conductance data with 1591 different antidot positions and 5 different random potentials. We checked that $\Delta G(B)$ shows standard mesoscopic conductance fluctuation with variance with the order of e^2/h.

Lines 195 – 196
(Added sentence)
We note that a part of wave function phase information is included in the WI because the amplitude is the results of complex interference between scattered waves.

Lines 200 – 203
(Added sentences)
A geometry image without WIs is defined as an image with a non-zero constant value on a nanowire and zero inside the antidots and outside the nanowire. No WI images are superimposed. The constant value is determined by the normalisation condition $\sum_{i=1}^{60}\sum_{j=1}^{60} X_{i,j} = 1$, where $\boldsymbol{X}$ is an image, and the suffixes i, j represent the pixel label.

Lines 208 – 209
(Added sentence)
The dimension of the latent space is set to 7 to reconstruct the input WI images with high accuracy.

Lines 210 – 212
(Added sentence)
The network architecture parameters are determined based on a recipe found in machine learning for image recognition[14] and then fine-tuned.

Lines 216 – 217
(Added sentence)
We used WI images as the inputs to the feature extraction network to extract defect position and quantum interference information.

Lines 229 – 233
(Added sentences)
Although reconstructing a Hamiltonian from wave functions (an inverse problem) is difficult, from a theoretical point of view, potential reconstruction is possible if scattering states are known for all the incident wavenumbers[23]. In the present case, although the incident wavenumbers are limited to those on the Fermi surface, the potential shape was estimated. This might be attributed to the fact that the potential shape is restricted to the antidot shape.

Lines 234 – 242
(Added sentences)
Latent-space dimension in QGD. To determine the appropriate dimension of the latent space, we performed control experiments. Supplementary Fig. 5 shows the latent-space dimension dependence of the averaged RMS error of the decoded WI images. For the feature extraction network, the latent-space dimension less than 5 was found to show large RMS errors, which can be attributed to insufficient capacity of the latent space as shown in Supplementary Fig. 5a, b. On the other hand, higher latent-space dimension than 9 was found to show large RMS errors due to too many learning parameters. Due to the competition between the two, the RMS error takes a minimum around at dimension = 7. A similar behavior was also found for the WI images decoded from the geometry generative network

(Supplementary Fig. 5c, d). Therefore, we determined to use the 7-dimensional latent space for the QGD network.

Lines 243 – 251
(Added sentences)
Generalisation ability of the QGD network. In Supplementary Figs. 7 and 8, we show some training results of the network with different tight-binding parameters: the Fermi energy and disorder potential, respectively. The result shows that the network we proposed exhibits high fidelity regardless of the parameters. Although the interference fringe patterns in the WI images largely depend on the Fermi energy and the disorder potential (see Supplementary Figs. 6 and 7 and those caption for details), the method can reconstruct WI images. In Supplementary Fig. 8, we also show the WI images decoded from the Fermi energy dependence of the magneto-conductance, where the Fermi energy is swept from $1.2t$ to $2.0t$ at zero magnetic field. The data shows that the WI images can also be decoded with high fidelity when we use the Fermi energy dependence data instead of the magnetic field dependence.

Lines 252 – 259
(Added sentences)
Quantitative analysis of the data structure in the latent space. We quantitatively estimated the difference between Fig. 3c and d by calculating the thicknesses of the data structures as the variance of the data points in the thickness direction. Firstly, as shown in Supplementary Fig. 11a, we cut out a part of the data structure in the latent space, which locally forms two-dimensional plane with a thickness. The local variance was calculated for the in-plane and thickness directions. The ratio of the local variance along the in-plane and thickness directions is 0.10 for the data structure formed by the geometry images with WIs [Supplementary Fig. 11c]. On the other hand, the data structure formed by the geometry images without WIs exhibits a much smaller value of the ratio, 0.01 [Supplementary Fig. 11b and d].

Lines 260 – 267
(Added sentences)
Dual-layered data structure in the latent space. In the present calculation, we chose the pixel size of the interference images so that the Fermi wavelength is equivalent to 2 pixels. In the condition, the data scattering in the latent space was found to apparently be quantised to show the most interpretable structure: the dual layers, which can be explained by the fact that a standing wave between an antidot and the sample end can be classified into two; destructive and constructive interference patterns. In fact, the dual-layer structure was found to change into a randomly scattered structure around the surface by changing the pixel size, showing that the essential feature here is the data scattering around the surface structure representing the information of the antidot positions.

Lines 268 – 274
(Added sentences)
Quantitative comparison between original and deciphered WI images. We analysed the WI images in terms of the normalized cross correlation:

$$R_{\mathrm{NCC}} = \frac{\sum_{i,j} A(i,j)B(i,j)}{\sqrt{\sum_{i,j} A(i,j)^2 \sum_{i,j} B(i,j)^2}}$$

, where A and B are the original and generated WI images, respectively. As shown in Supplementary Fig. 9a, b, R_{NCC} increases with the training epoch. R_{NCC} after the training is

much greater ($R_{\text{NCC}} > 0.997$) than that before the training. We confirmed that the generated WI images show high fidelity in terms of the normalised cross correlation.

Figures

We rearranged Fig. 3 and Fig. 4 and revised the figure labels

We revised the figure panels in Figs. 1-4 and Supplementary Fig. 3.

We revised the figure captions in Figs. 1-4 and Supplementary Fig. 3.

We revised the labels of the axes and the color code in Figs. 1-4 and Supplementary Figs. 3 and 4

We revised the unit of the *x*-axis in Fig. 1c

We added a scale bar to Fig. 4e.

We added Supplementary Fig. 5-11 to Supplementary Information.

REVIEWER COMMENTS

Reviewer #1 (Remarks to the Author):

The authors clarified properly almost all comments in the review reports. In the revised manuscript, it is necessary to correct the wording "electron wave function" in Fig. 1(b).

Reviewer #2 (Remarks to the Author):

Report on the revised manuscript by Dr Daimon and co-workers entitled "Deciphering Quantum Fingerprints in Electric Conductance" NCOMMS-20-50538

The manuscript has certainly improved, but largley due to the many edits requested by all reviewers. Usually, such a large number of changes suggests that the original manuscript was not yet ready for submission and I am always wary about authors making use of reviewers for what should have really been their own, internal quality check.

Let me, in this 2nd round of reviews, concentrate on the points that I raised before.

[2-3b]: The authors still claim that the thickness in latent space is due to "non-local" quantum interference. Indeed, they label said thickness in Fig. 3 with a red arrow denoted "quantum correction". If that was the case, I would expect that upon \hbar->0, this should vanish. However, such a check has not been made.

Furthermore, I would suggest that the observed thickness is simply related to the strength of the WI variation. Clearly, this will not happen for the chosen comparison when the intensities are ignored. However, if one would, for example, induce a small potential variation (smooth or random), this would even within classical and/or Boltzmann transport lead to a variation in electron intensity (= local density). I would speculate that in that case, a finite thickness can also be observed, but of course unrelated to any "quantumness".

Let me rephrase: the observed thickness is induced by the intensity variations. These in turn can be caused by many different physical phenomena of which "quantum interference" is one, but not the only one. If the authors agree, they should reformulate such statements in the text, for exmaple lines 145-146 and 159, among others.

["Answer 2-4"]: I do not understand what is meant by "the potential shape was estimated".

[2-6] line 80, "to 60 × 60 pixel data, and zero padding" suggest 60 x 65 to me.

I suggest to write "Each 60 x 50 WI images has added a zero padding of width 5 applied to the left and right ends of the nanowire to have a final size of 60x 60." or some such more precise statement.

A few additional comments:

[line 46] "extreme complexity": I don't think that the term "extreme" is needed or correct (what measure of "extreme complexity" is being implied?) and should be deleted.

[line 118] RMS error should have a unit. Isn't it in units of |psi^2|?

[line 121] "the 7-dimensional data in the latent space ... smaller than the dimension of the WI image: 3600". The "dimension" of the images is 2D, not 3600. So there is a mix of language between ML and physics. I suggest to correct this in a consistent way for the reader.

Reviewer #4 (Remarks to the Author):

Having read through the three reviews as well as the revised manuscript, I think that the authors have done greatly in addressing the referees' feedback. The only two comments I want to make about the revised manuscript are as follows:

1. I think that Figures R1a, R2, and R3, along with the corresponding discussions that are part of the rebuttal are too important to not be included into the manuscript (perhaps in Supplementary Information). On a related minor note, the manuscript would also benefit from having reference to "Lee, P. A., & Ramakrishnan, T. V. Disordered electronic systems. Rev. Mod. Phys. 57, 287-337 (1985)" cited in answer 2-3a to be added to it.

2. The name of the parameter "t" used in the Hamiltonian should be unified, as it is referred to as "hopping parameter" on lines 177-178 and "hopping energy" in the rest of the paragraph.

[Reply to the comment of Referee #1]
We appreciate the referee for the valuable time spent on the evaluation of our manuscript and the positive comment. We have carefully revised the manuscript in accordance with the referee's comment. The authors' reply to the comment and the revised part of the manuscript are detailed below.

(Comment 1-1)
The authors clarified properly almost all comments in the review reports. In the revised manuscript, it is necessary to correct the wording "electron wave function" in Fig. 1(b).

(Answer 1-1)
Thank you very much for the comment. Following the referee's comment, we revised the wording "electron wave function" to "electron wave function intensity" in Fig. 1b.

[Reply to the comments of Referee #2]

We thank the referee for the valuable time spent on the evaluation of our manuscript. Based on the suggestions, we have carefully revised the manuscript. In the following, we reply to the referee's insightful questions and comments.

(Comment 2-1)

[2-3b]: The authors still claim that the thickness in latent space is due to "non-local" quantum interference. Indeed, they label said thickness in Fig. 3 with a red arrow denoted "quantum correction". If that was the case, I would expect that upon ¥hbar->0, this should vanish. However, such a check has not been made.

Furthermore, I would suggest that the observed thickness is simply related to the strength of the WI variation. Clearly, this will not happen for the chosen comparison when the intensities are ignored. However, if one would, for example, induce a small potential variation (smooth or random), this would even within classical and/or Boltzmann transport lead to a variation in electron intensity (= local density). I would speculate that in that case, a finite thickness can also be observed, but of course unrelated to any "quantumness".

Let me rephrase: the observed thickness is induced by the intensity variations. These in turn can be caused by many different physical phenomena of which "quantum interference" is one, but not the only one. If the authors agree, they should reformulate such statements in the text, for exmaple lines 145-146 and 159, among others.

(Answer 2-1)

Thank you very much for the comments. Following the referee's suggestion, we have revised the sentences in the main text (Lines 146 – 147, 160 – 161, 161 – 163, 168 – 169, Page 6; Lines 218 – 219, Page 8). The original and revised sentences are as follows:

Lines 146 – 147, Page 6

(Original manuscript)

The result suggests that the thickness structures, such as the dual-layered structure, is due to the quantum interference of wave functions.

(Revised manuscript)

The result suggests that the thickness structures, such as the dual-layered structure, carry electron information represented by WIs.

Lines 160 – 161, Page 6

(Original manuscript)

… suggesting that the constructive and deconstructive interference gives rise to the observed dual-layered structure in the latent space (see Methods for more details).

(Revised manuscript)

… suggesting that, in the present study, the constructive and deconstructive interference gives rise to the observed dual-layered structure in the latent space (see Methods for more details).

Lines 161 – 163, Page 6

(Original manuscript)

The result shows that the effect of quantum interference, quantum WI correction to the conductivity beyond the standard Boltzmann transport, is encoded in the latent space as the

thickness-dimension information, which emerges on the 2-dimensional curved surface representing the classical information.

(Revised manuscript)
The result suggests that the electron information, such as interference, is encoded in the latent space as the thickness-dimension information, which emerges on the 2-dimensional curved surface representing the antidot location.

Lines 168 – 169, Page 6
(Original manuscript)
The quantum interference turned out to be encoded in the latent space of QGD as an extra dimension of the manifold representing the classical information.

(Revised manuscript)
The WI patterns turned out to be encoded in the latent space of QGD as an extra dimension of the manifold representing the defect position information.

Lines 218 – 219, Page 8
(Original manuscript)
We used WI images as the inputs to the feature extraction network to extract defect position and quantum interference information.

(Revised manuscript)
We used the WI images as the inputs to the feature extraction network to extract defect position and WI information.

We have also revised the caption to Fig. 1 and the label "quantum correction" in Fig. 3c to "thickness".

(Comment 2-2)
["Answer 2-4"]: I do not understand what is meant by "the potential shape was estimated".

(Answer 2-2)
We thank the referee for the question. In the previous response letter, we used the sentence *"the potential shape was estimated"* to mean that the spatial distribution of the potential (antidot position) was reproduced. We have revised the sentence in Methods (Lines 235 – 237, Page 9).

(Comment 2-3)
[2-6] line 80, "to 60 × 60 pixel data, and zero padding" suggest 60 x 65 to me.
I suggest to write "Each 60 x 50 WI images has added a zero padding of width 5 applied to the left and right ends of the nanowire to have a final size of 60x 60." or some such more precise statement.

(Answer 2-3)
Thank you very much for the comments. Following the suggestion, we have revised the sentence in the main text (Lines 82 – 83, Page 3).

(Comment 2-4)
[line 46] "extreme complexity": I don't think that the term "extreme" is needed or correct (what measure of "extreme complexity" is being implied?) and should be deleted.

(Answer 2-4)
Thank you for the comment. Following the referee's instruction, we have removed the term "extreme" in the main text (Line 48, Page 2).

(Comment 2-5)
[line 118] RMS error should have a unit. Isn't it in units of |psi^2|?

(Answer 2-5)
Thank you for the comment. The RMS error between two WI images is a dimensionless quantity in the present study. It can be confirmed because the WI images **X** and **Y** are dimensionless quantities that satisfy the normalization conditions: $\sum_{i=1}^{60}\sum_{j=1}^{60} X_{i,j} = 1$ and $\sum_{i=1}^{60}\sum_{j=1}^{60} Y_{i,j} = 1$. To clarify the point, in response to the referee's comment, we have added sentences to Methods (Lines 222 – 224, Page 8).

(Comment 2-6)
[line 121] "the 7-dimensional data in the latent space ... smaller than the dimension of the WI image: 3600". The "dimension" of the images is 2D, not 3600. So there is a mix of language between ML and physics. I suggest to correct this in a consistent way for the reader.

(Answer 2-6)
Thank you for the comment. Following the referee's suggestion, we have revised the sentence in the main text (Line 123, Page 5).

[Reply to the comments of Referee #4]
We thank the referee for the valuable time spent on the evaluation of our manuscript and positive comments. Following the comments, we have carefully revised the manuscript. In the following, we reply to the referee's fruitful comments.

(Comment 4-1)
I think that Figures R1a, R2, and R3, along with the corresponding discussions that are part of the rebuttal are too important to not be included into the manuscript (perhaps in Supplementary Information).

(Answer 4-1)
Thank you for the comment. Following the comment, we have added Figs. R1, R2, and R3 in the previous response letter to Supplementary information as Supplementary Figs. 12, 13, and 14, respectively.

(Comment 4-2)
On a related minor note, the manuscript would also benefit from having reference to "Lee, P. A., & Ramakrishnan, T. V. Disordered electronic systems. Rev. Mod. Phys. 57, 287-337 (1985)" cited in answer 2-3a to be added to it.

(Answer 4-2)
Thank you for the comment. Following the comment, we have added the reference [Lee, P. A. & Ramakrishnan, T. V. Disordered electronic systems. *Rev. Mod. Phys.* **57**, 287-337 (1985).] to the revised manuscript (Line 46, Page 2).

(Comment 4-3)
The name of the parameter "t" used in the Hamiltonian should be unified, as it is referred to as "hopping parameter" on lines 177-178 and "hopping energy" in the rest of the paragraph.

(Answer 4-3)
We thank the referee for the comment. Following the referee's comment, we have unified the name of "*t*" used in the Hamiltonian to "hopping energy" (Lines 179 and 188, Page 7).

[List of corrections]

Main text

Line 48, Page 2
(Original manuscript)
Nevertheless, it has been considered difficult to interpret it due to its extreme complexity.

(Revised manuscript)
Nevertheless, it has been considered difficult to interpret it due to its complexity.

Lines 82 – 83, Page 3
(Original manuscript)
Each WI image is converted to 60 × 60 pixel data, and zero padding[15] of 5 pixels was applied to the left and right ends of the nanowire.

(Revised manuscript)
Zero padding[16] with a width of 5 pixels is added to the left and right ends of each 60 × 50 pixel WI image, resulting in the final size of 60 × 60 pixels.

Line 123, Page 5
(Original manuscript)
… which is three orders of magnitude smaller than the dimension of the WI image: 3600.

(Revised manuscript)
… which is three orders of magnitude smaller than the dimension of the input data: 3600.

Lines 133 – 134, Page 5
(Original manuscript)
Owing to the variational Bayesian approach of VAE[13], VAE was found to construct well-organised data structures in the latent space.

(Revised manuscript)
Owing to the variational Bayesian approach of VAE[14], VAE was found to construct well-organised data structures in the latent space (see also Supplementary Fig. 12).

Lines 146 – 147, Page 6
(Original manuscript)
The result suggests that the thickness structures, such as the dual-layered structure, is due to the quantum interference of wave functions.

(Revised manuscript)
The result suggests that the thickness structures, such as the dual-layered structure, carry electron information represented by WIs.

Lines 160 – 161, Page 6
(Original manuscript)
… suggesting that the constructive and deconstructive interference gives rise to the observed dual-layered structure in the latent space (see Methods for more details).

(Revised manuscript)
… suggesting that, in the present study, the constructive and deconstructive interference gives rise to the observed dual-layered structure in the latent space (see Methods for more details).

Lines 161 – 163, Page 6
(Original manuscript)
The result shows that the effect of quantum interference, quantum WI correction to the conductivity beyond the standard Boltzmann transport, is encoded in the latent space as the thickness-dimension information, which emerges on the 2-dimensional curved surface representing the classical information.

(Revised manuscript)
The result suggests that the electron information, such as interference, is encoded in the latent space as the thickness-dimension information, which emerges on the 2-dimensional curved surface representing the antidot location.

Lines 168 – 169, Page 6
(Original manuscript)
The quantum interference turned out to be encoded in the latent space of QGD as an extra dimension of the manifold representing the classical information.

(Revised manuscript)
The WI patterns turned out to be encoded in the latent space of QGD as an extra dimension of the manifold representing the defect position information.

Methods

Lines 179 and 188, Page 7
We have unified the name of "*t*" used in the Hamiltonian to "hopping energy".

Lines 195 – 196, Page 7
(Original manuscript)
We checked that $\Delta G(B)$ shows fluctuation with respect to *B* with the variance comparable to e^2/h[18].

(Revised manuscript)
We checked that the conductance calculation works well (see Supplementary Figs. 13 and 14) and $\Delta G(B)$ shows fluctuation with respect to *B* with the variance comparable to e^2/h[19].

Lines 218 – 219, Page 8
(Original manuscript)
We used WI images as the inputs to the feature extraction network to extract defect position and quantum interference information.

(Revised manuscript)
We used the WI images as the inputs to the feature extraction network to extract defect position and WI information.

Lines 222 – 224, Page 8
(Added manuscript)
The RMS error is a dimensionless quantity because the WI images $\mathbf{X}^{(n)}$ and $\mathbf{Y}^{(n)}$ are dimensionless quantities that satisfy the normalization conditions: $\sum_{i=1}^{60}\sum_{j=1}^{60} X_{i,j}^{(n)} = 1$ and $\sum_{i=1}^{60}\sum_{j=1}^{60} Y_{i,j}^{(n)} = 1$.

Lines 235 – 237, Page 9
(Original manuscript)
In the present case, although the incident wavenumbers are limited to those on the Fermi surface, the potential shape was estimated.

(Revised manuscript)
In the present case, although the incident wavenumbers are limited to those on the Fermi surface, the spatial distribution of the potential is reproduced.

Figures

We revised the figure panels in Figs. 1b and 3c.

We revised the caption to Fig. 1.

We added Supplementary Figs. 12-14 to Supplementary Information.

References

We have added the reference 8 [Lee, P. A. & Ramakrishnan, T. V. Disordered electronic systems. *Rev. Mod. Phys.* **57**, 287-337 (1985)] to the main text.
