# Supplementary material for: Deciphering quantum fingerprints in electric conductance
Source: Nat Commun. 2022 Jun 8;13:3160. doi: 10.1038/s41467-022-30767-w (PMC9177777; doi:10.1038/s41467-022-30767-w)
Supplement: Supplementary file 2 — Peer Review File [file 41467_2022_30767_MOESM2_ESM.pdf]

Supplementary Information

Deciphering quantum fingerprints in electric conductance

S. Daimon, K. Tsunekawa, S. Kawakami, T. Kikkawa, R. Ramos, K. Oyanagi, T. Ohtsuki & E. Saitoh

Table of Contents

Supplementary Figure 1 | Architecture of the feature extraction and geometry generative networks.

Supplementary Figure 2 | Learning curves.

Supplementary Figure 3 | Autoencoding of geometry images using the feature extraction network after training.

Supplementary Figure 4 | An experiment using a real nano system.

Supplementary Figure 5 | The latent-space dimension dependence of the training results.

Supplementary Figure 6 | Result of Quantum Geometric Decoding using data with different Fermi energy.

Supplementary Figure 7 | Result of Quantum Geometric Decoding using data with greater disorder potentials.

Supplementary Figure 8 | Result of Quantum Geometric Decoding using the Fermi-energy dependence of conductance.

Supplementary Figure 9 | Quantitative analysis of the deciphered WI images.

Supplementary Figure 10 | The position dependence of the absolute difference between the original and deciphered geometry images.

Supplementary Figure 11 | Dispersion analysis on the data generated from geometry images with and without the WI information.

Supplementary Figure 12 | Principal component analysis (PCA) on the conductance traces.

Supplementary Figure 13 | Convergence of the conductance data.

Supplementary Figure 14 | Comparison between the calculated magnetic fingerprints with opposite magnetic field directions.

Supplementary Table 1 | Architecture of the convolution part of the feature extraction network.

Supplementary Table 2 | Architecture of the deconvolution part of the feature extraction network.

Supplementary Table 3 | Architecture of the prediction part of the geometry generative network.

Supplementary Note 1 | Experimental.

Supplementary References

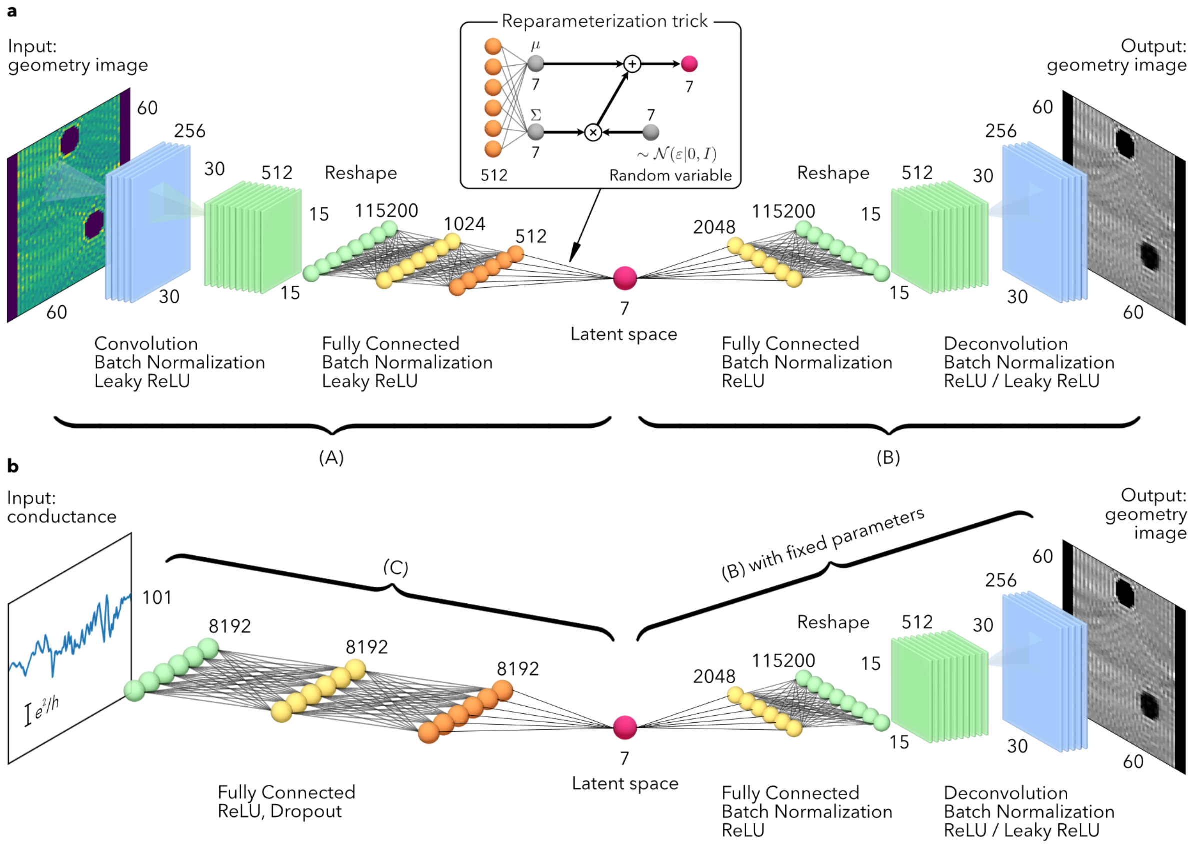

Supplementary Figure 1 | Architecture of the feature extraction and geometry generative networks. a The architecture of the feature extraction network based on the variational autoencoder[1]. The feature extraction network consists of a convolution part and a deconvolution part, labeled as (A) and (B), respectively. The convolution part is composed of 2 two-dimensional convolution layers and 3 fully-connected layers (see Supplementary Table 1 for more details). The input mono-channel image size is 60 $\times$ 60, and the output is the mean value $\boldsymbol{\mu}$ and the logarithm variance $\boldsymbol{\Sigma}$ in the 7-dimensional latent space. A 7-dimensional vector in the latent space is randomly selected in the reparameterization trick[1,2]. The deconvolution part is composed of 2 fully-connected layers and 2 two-dimensional transposed convolution layers (see Supplementary Table 2 for more details). The input is a 7-dimensional vector in the latent space, and the output mono-channel image size is 60 $\times$ 60. We used rectified linear units (ReLUs)[3] or leaky ReLUs[4] for activation functions and the batch normalisation technique[5] in the feature extraction network. The input wave function images are multiplied by 5 as a preprocess for a training. **b** The architecture of the geometry generative network. The geometry generative network consists of a prediction part and the deconvolution part, labeled as (C) and (B), respectively. The prediction part is composed of 4 fully-connected layers with ReLU activation functions and the dropout technique[6] (see Supplementary Table 3 for more details). The

input is normalised magneto-conductance data with 101 points, and the output is a 7-dimensional vector in the latent space. The deconvolution part is the same as that of the feature extraction network.

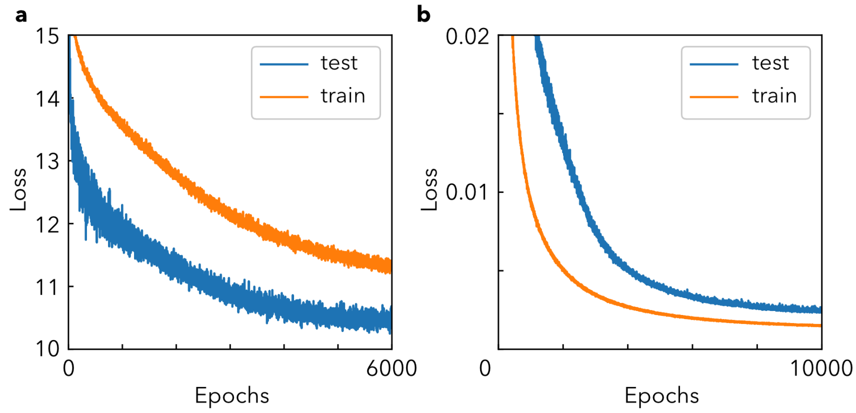

Supplementary Figure 2 | Learning curves. a Loss values for the training and test datasets at each epoch are plotted for the feature extraction network. The network is trained by the adaptive moment estimation[7] (Adam) with the beta hyperparameters of (0.5, 0.999) and the learning rate of 0.0001. The loss function is the evidence lower bound[1] $L = \frac{1}{N}\sum_{n=1}^{N}\{\mathrm{SE}_n + D_{\mathrm{KL}}[N(\mathbf{z};\ \boldsymbol{\mu}_n, \boldsymbol{\Sigma}_n)\ ||\ N(\mathbf{z};\ \mathbf{0},\ \mathbf{I})]\}$, where n is the sample labeling number, N is the number of the samples in the dataset, SE_n is the squared error between the n-th input image $\mathbf{X}^{(n)}$ and the n-th output image $\mathbf{Y}^{(n)}$; $\mathrm{SE}_n = \sum_{i=1}^{60}\sum_{j=1}^{60}\left[X_{i,j}^{(n)} - Y_{i,j}^{(n)}\right]^2$, the suffixes i, j representing the pixel, D_{KL} is the Kullback-Leibler divergence, $N(\mathbf{z}; \boldsymbol{\mu}, \boldsymbol{\Sigma})$ is a Gaussian distribution with its mean value $\boldsymbol{\mu}$ and covariance matrix $\boldsymbol{\Sigma}$, and $\mathbf{I}$ is the identity matrix. Batch size for the training is 256. The test loss is less than the training loss since the reparameterization trick is not used for the test loss evaluation. **b** Loss values for the training and test datasets at each epoch are plotted for the geometry generative network. The network is trained by the adaptive moment estimation[7] (Adam) with the beta hyperparameters of (0.5, 0.999) and the learning rate of 0.00001. The loss function L is the mean squared error of the points in the latent space; $L = \frac{1}{N}\sum_{n=1}^{N}\left\|\mathbf{z}^{(n)} - \mathbf{y}^{(n)}\right\|^2$, where N is the number of samples in the dataset, $\mathbf{y}^{(n)}$ is the output of the network (C) [Supplementary Fig. 1b] from the n-th magneto-conductance input, $\mathbf{z}^{(n)}$ is the outputs of the trained network (A) [Supplementary Fig. 1a] from the n-th wave function image input. The batch size for the training is 256. The dropout technique is not used for the test loss evaluation.

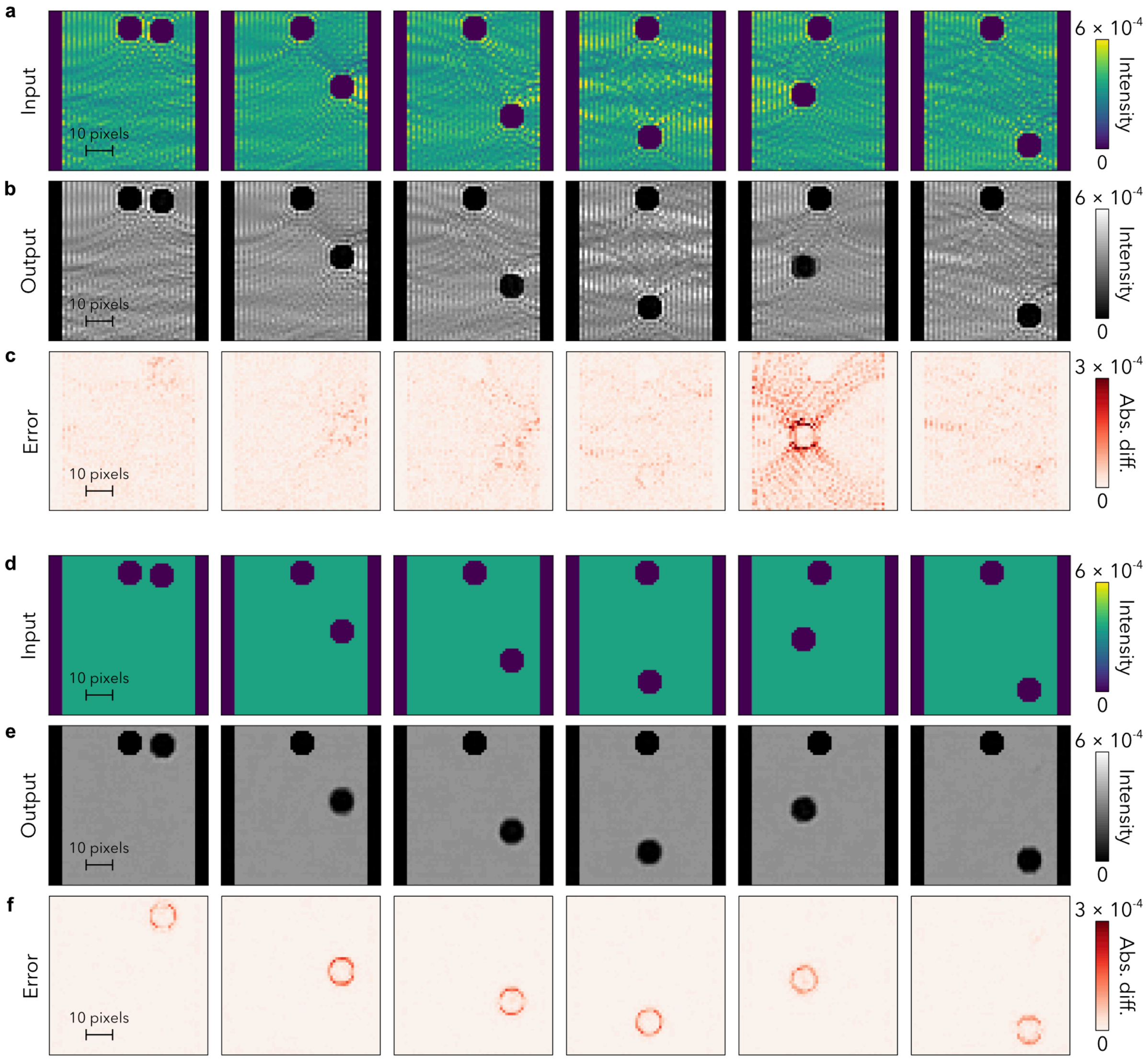

Supplementary Figure 3 | Autoencoding of geometry images using the feature extraction network after training. Examples of the input, output, and error images for the feature extraction network (Supplementary Fig. 1a), trained by the geometry images with wave function intensities (WIs) (**a**, **b**, **c**) and without WIs (**d**, **e**, **f**). The input images are randomly selected from the test dataset, not used for the training of the network. The error is defined as the absolute difference between the values of each pixel in the original and output images.

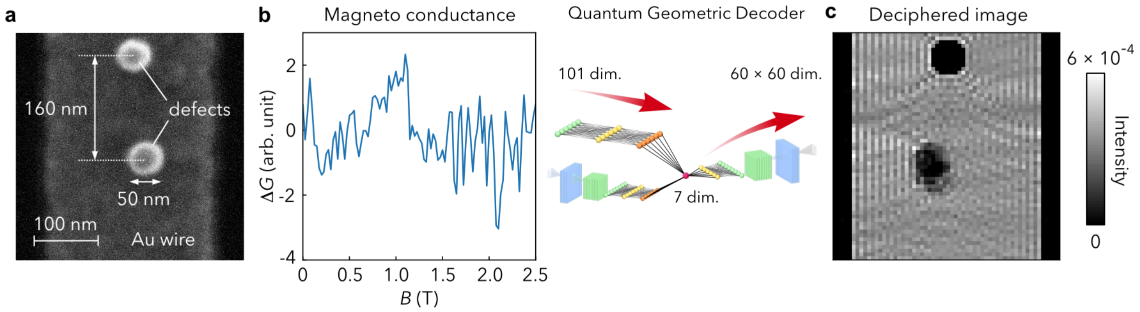

Supplementary Figure 4 | An experiment using a real nano system. a A scanning electron microscope image of an Au wire fabricated by electron beam lithography. Black and gray areas are the SiO_2 substrate and Au wire, respectively. The white circles in the wire are the damaged areas introduced by electron beam exposure. **b** The magnetic field B dependence of the normalised magneto-conductance ΔG measured by a four probe measurement at 100 mK (see Supplementary Note 1 for details). **c** The output of the QGD network for the data in **b**.

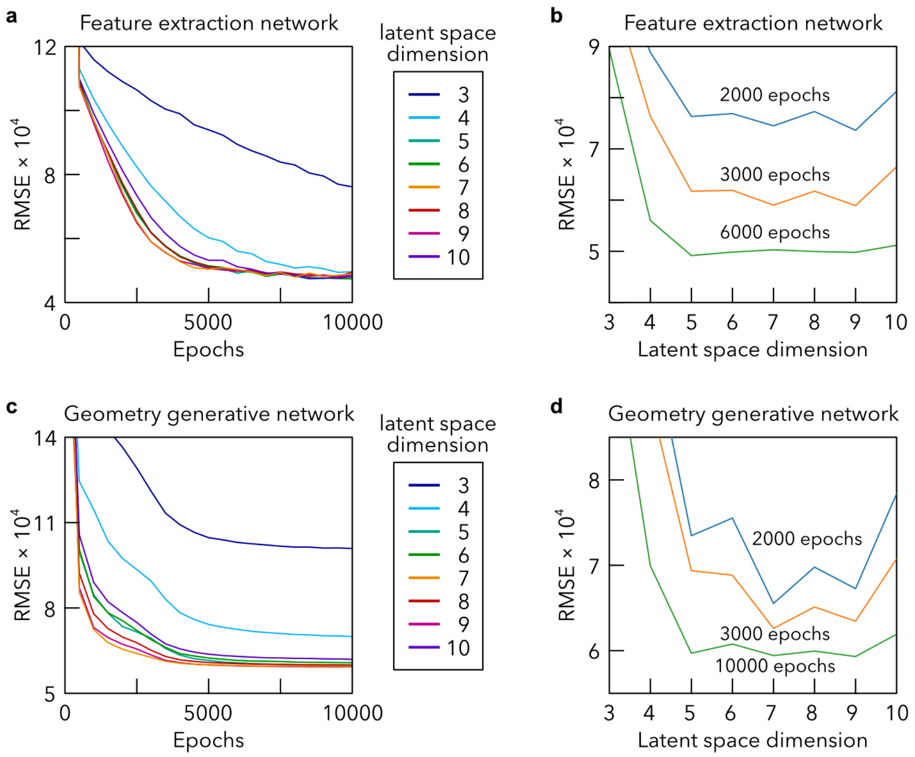

Supplementary Figure 5 | The latent-space dimension dependence of the training results. a The training epoch dependence of the RMS error between the original and deciphered images generated from the feature extraction network. The training results for different latent-space dimensions are plotted by different colours. **b** The latent-space dimension dependence of the RMS error between the original and deciphered images generated from the feature extraction network. The training results for 2000, 3000, and 6000 epochs are plotted by blue, orange, and green lines. **c** The training epoch dependence of the RMS error between the original and deciphered images generated via the geometry generative network. The training results for different latent-space dimensions are plotted by different colours. **d** The latent-space dimension dependence of the RMS error between the original and deciphered images generated via the geometry generative network. The training results for 2000, 3000, and 10000 epochs are plotted by blue, orange, and green lines.

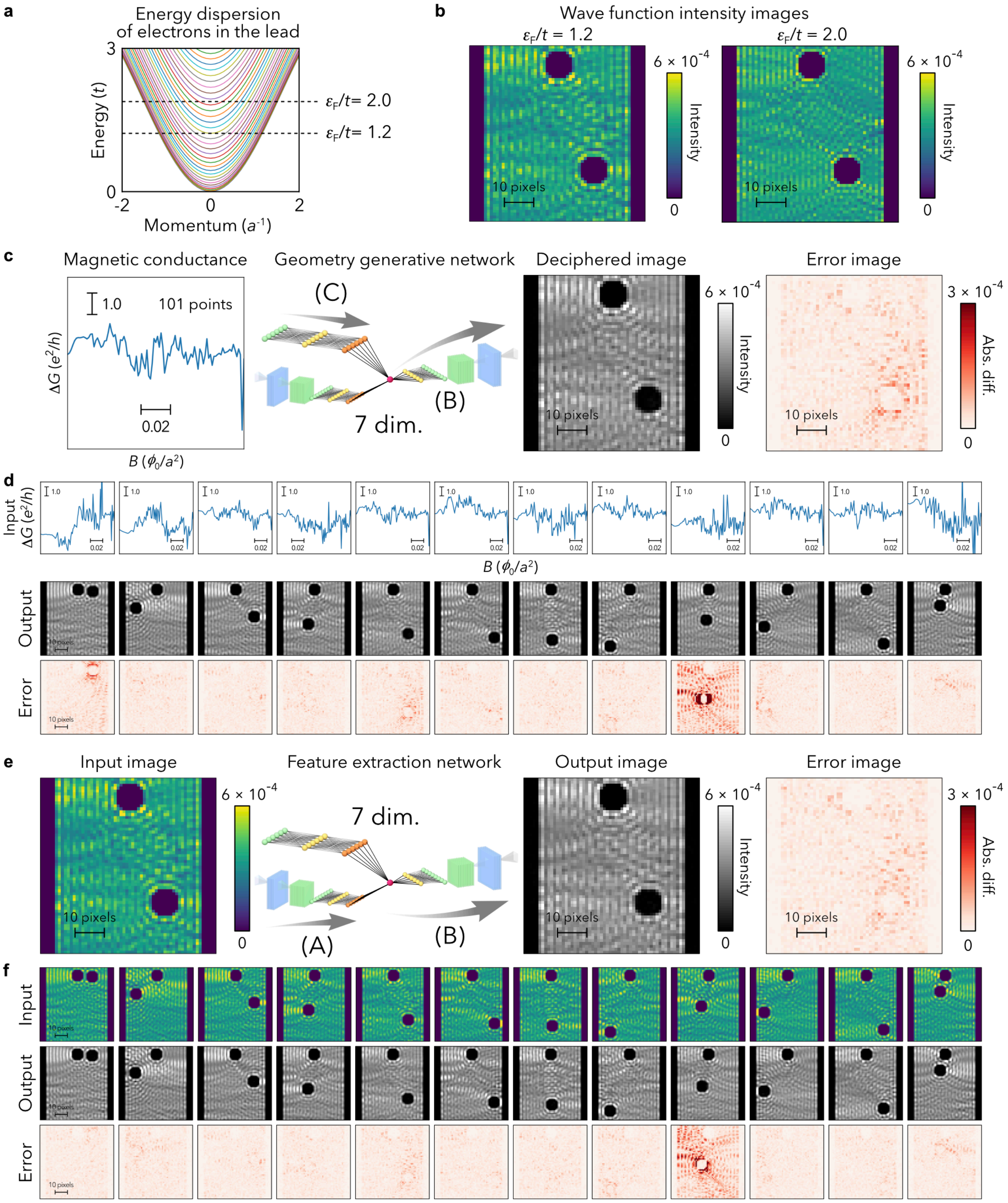

Supplementary Figure 6 | Result of Quantum Geometric Decoding using data with different Fermi energy. **a** The energy dispersion relation of electrons in the lead. t, ε_{F}, and a are the hopping energy, Fermi energy, and lattice constant, respectively. **b** Wave function intensity images for $\varepsilon_{\mathrm{F}}/t = 1.2$ and 2.0. We used $\varepsilon_{\mathrm{F}}/t = 1.2$ for the machine learning shown in **c-f** while we used $\varepsilon_{\mathrm{F}}/t = 2.0$ for the experiments in the main text. **c** The geometry generative network and an example of the input conductance, output deciphered image, and absolute difference between the original and deciphered images. ΔG and B are the normalised magneto-

conductance and the magnetic flux density, respectively. $\phi_0 = h/e$ is the magnetic flux quantum. **d** Examples of the input conductance, output deciphered images, and error images. **e** The feature extraction network and an example of the input WI image, output deciphered image, and absolute difference between the original and deciphered images. **f** Examples of the input images, output deciphered images, and error images.

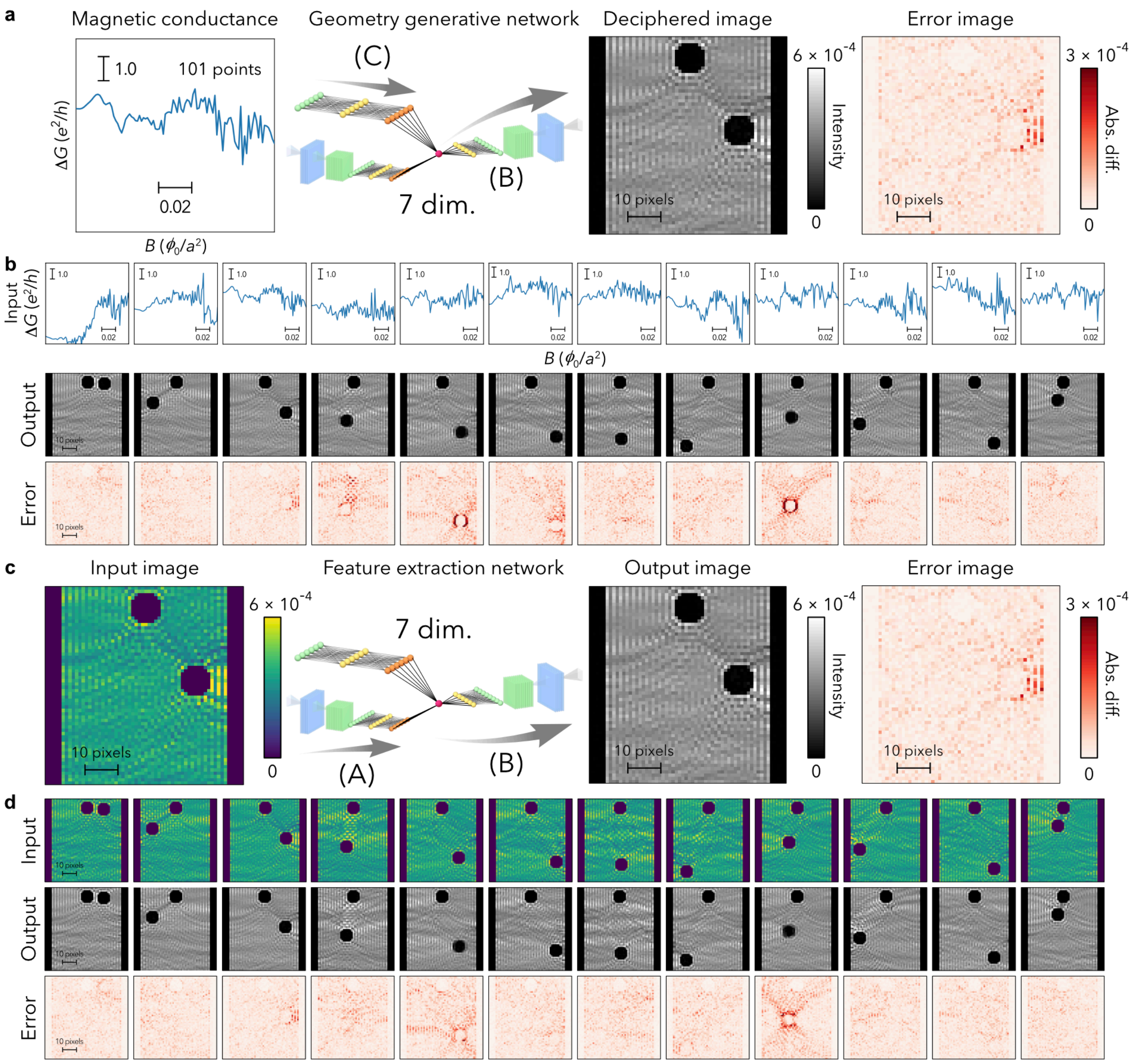

Supplementary Figure 7 | Result of Quantum Geometric Decoding using data with greater disorder potentials. a The geometry generative network and an example of the input conductance, output deciphered image, and absolute difference between the original and deciphered images. Here, the disorder-potential amplitude is doubled compared to that used for the dataset preparation in the main text. **b** Examples of the input conductance, output deciphered images, and error images. **c** The feature extraction network and an example of the input WI image, output deciphered image, and absolute difference between the original and deciphered images. **d** Examples of the input images, output deciphered images, and error images.

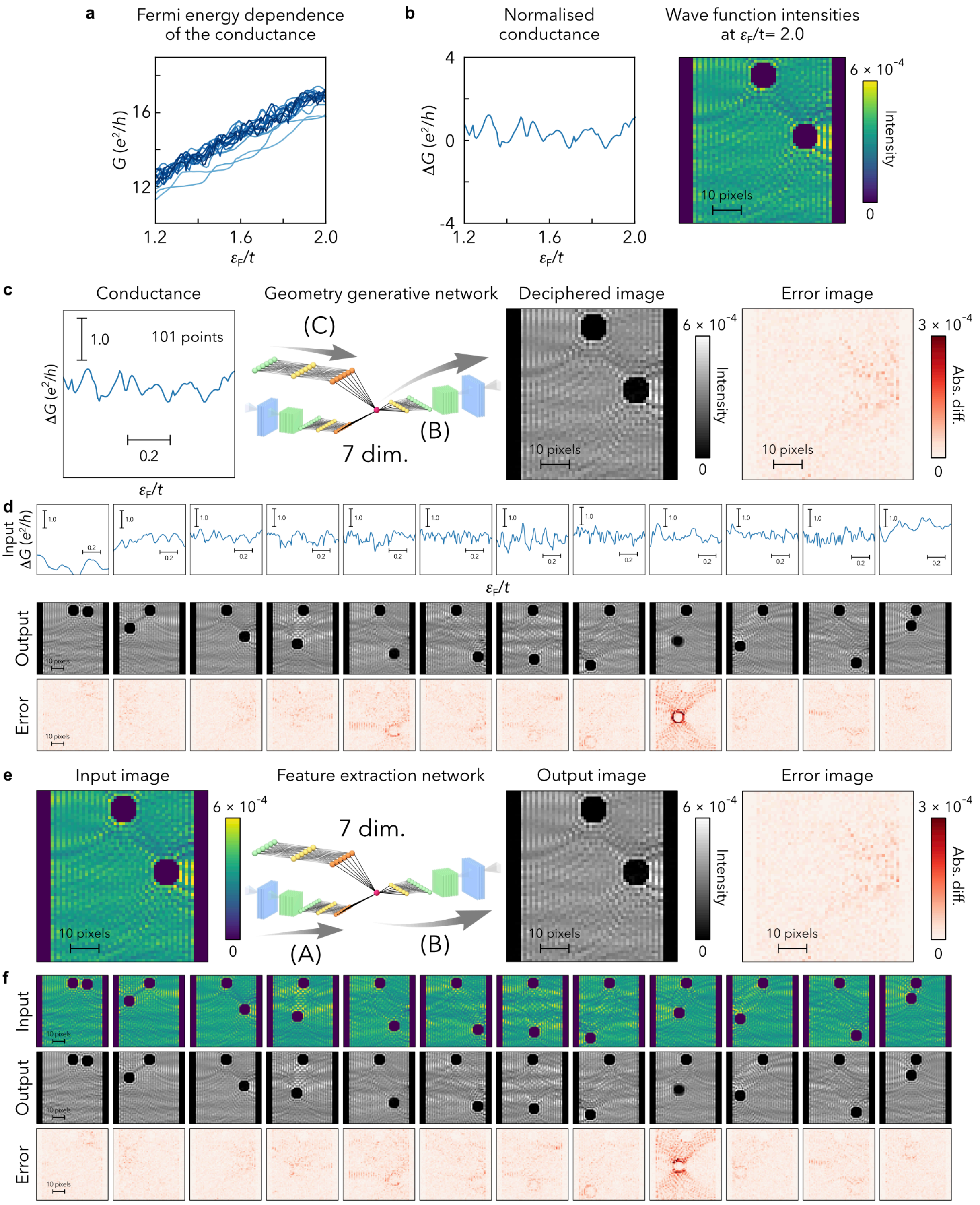

Supplementary Figure 8 | Result of Quantum Geometric Decoding using the Fermi-energy dependence of conductance. a The Fermi energy dependence of the conductance at zero magnetic field and $\varepsilon_{\mathrm{F}}/t = 2.0$. **b** Normalised conductance and a WI image used for the training. **c** The geometry generative network and an example of the input conductance, output deciphered image, and absolute difference between the original and deciphered images. **d** Examples of the input conductance, output deciphered images, and error images.

e The feature extraction network and an example of the input WI image, output deciphered image, and absolute difference between the original and deciphered images. **f** Examples of the input images, output deciphered images, and error images.

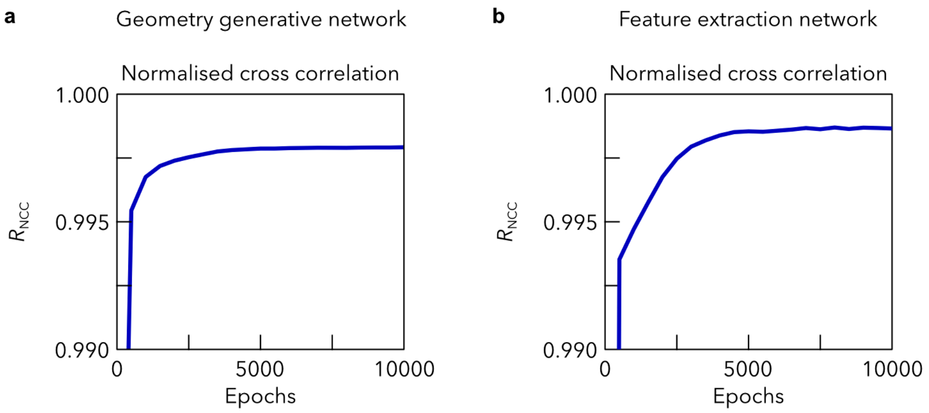

Supplementary Figure 9 | Quantitative analysis of the deciphered WI images. a, b The training epoch dependence of the normalised cross correlation:

$$R_{\mathrm{NCC}} = \frac{\sum_{i,j} A(i,j)B(i,j)}{\sqrt{\sum_{i,j} A(i,j)^2 \sum_{i,j} B(i,j)^2}}$$

between the original WI image (**A**) and the deciphered WI image (**B**) for the geometry generative network (**a**) and the feature extraction network (**b**). We confirmed that the generated WI images show high fidelity (R_{NCC} > 0.997) in terms of the normalised cross correlation.

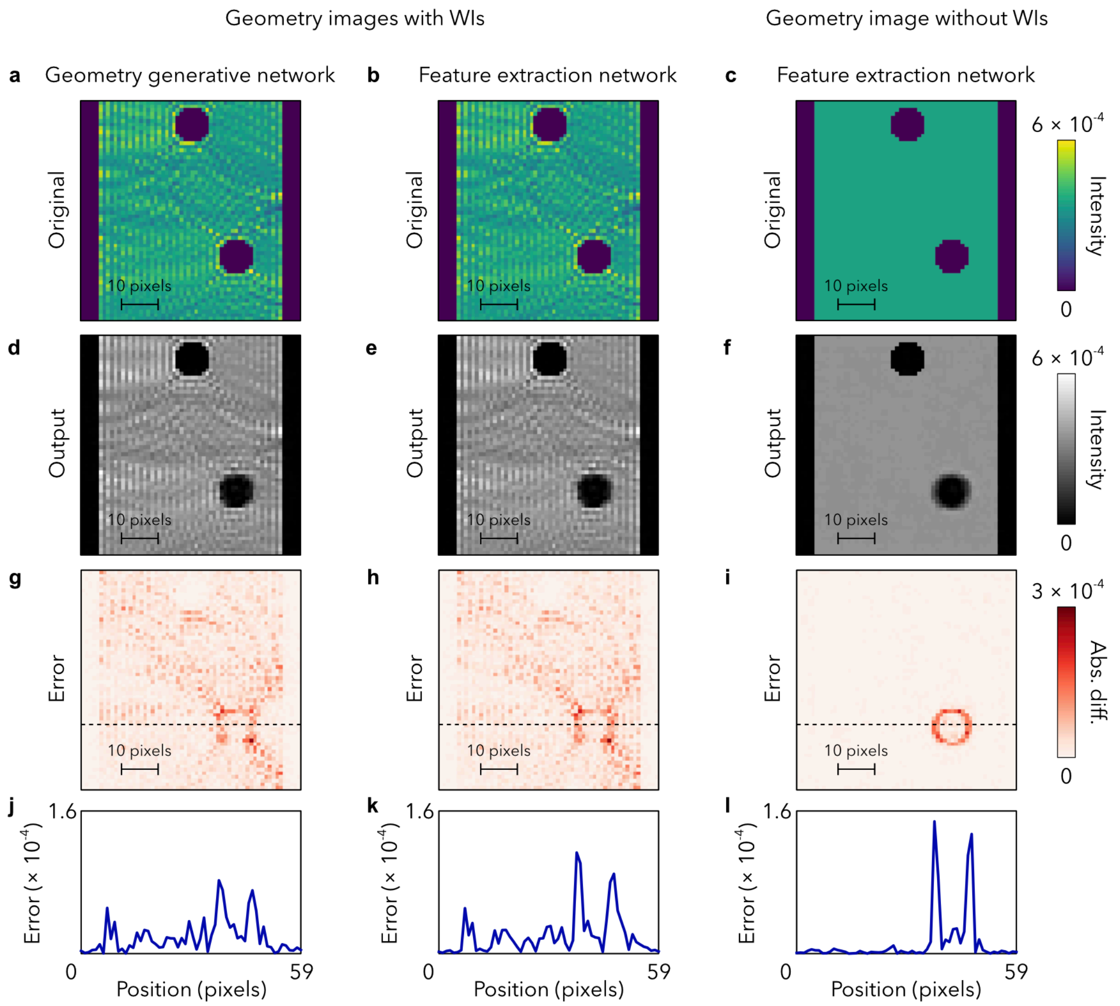

Supplementary Figure 10 | The position dependence of the absolute difference between the original and deciphered geometry images. a, **b**, **c** The input geometry images with WIs for the geometry generative network (**a**) and the feature extraction network (**b**), and the input geometry image without a WI for the feature extraction network (**c**). **d**, **e**, **f** The corresponding output geometry images. **g**, **h**, **i** The absolute difference (error) images between the input and output images corresponding to the data in **a**, **b**, and **c**. **j**, **k**, **l** The one-dimensional position dependence of the absolute difference (error) between the input and output images. In the panels **j**, **k**, and **l**, we plotted the error values along the dotted lines in **g**, **h**, and **i**, respectively.

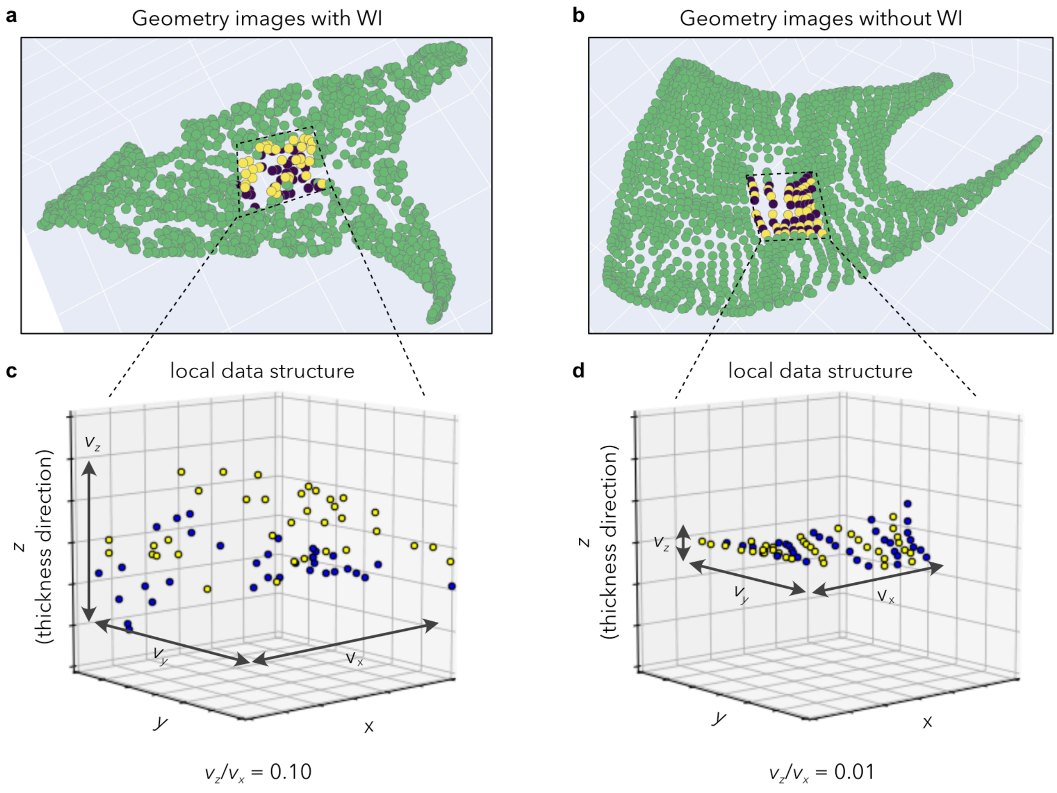

Supplementary Figure 11 | Dispersion analysis on the data generated from geometry images with and without the WI information. a, b Data structures generated in the latent space trained with the geometry images together with WIs (**a**), and without WIs (**b**). **c, d** Local structures cut out from the data shown in **a** and **b**, respectively. The *x*, *y*, and *z* axes are chosen so as to maximise v_x and then maximise v_y, where v_x, v_y, and v_z are the local variance of the data structure along the *x*, *y*, and *z* axes, respectively. v_z/v_x are 0.10 and 0.01 for the data structure in **c** and **d**, respectively.

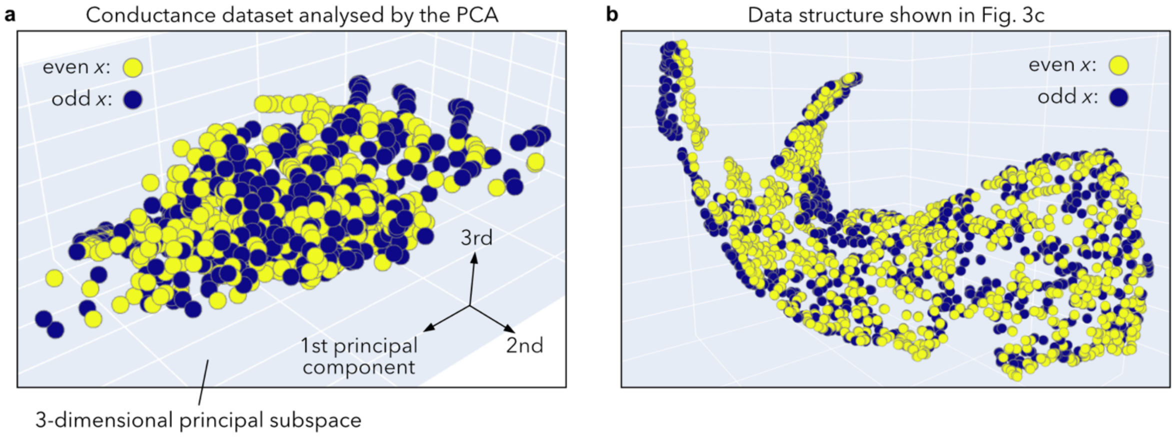

Supplementary Figure 12 | Principal component analysis (PCA) on the conductance traces. a Output from the PCA analysis on the conductance plotted in the 3-dimensional principal subspace. **b** Data structure shown in Fig. 3c (output of the feature extraction network).

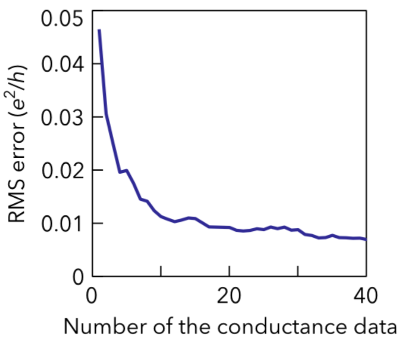

Supplementary Figure 13 | Convergence of the conductance data. We calculated 500 conductance traces with different disorder configurations with fixed antidot positions and obtained the averaged conductance $\mathbf{G}_{\mathrm{AVE}}$. To check the convergence of the averaged conductance, we plotted the RMS error: $\sqrt{\sum_{i=1}^{101}\left|G_{n,i} - G_{\mathrm{AVE},i}\right|^2/101}$, where n is the number of the disorder configurations and $\mathbf{G}_n$ is the disorder averaged conductance averaged over n conductance traces. i is the label representing the magnetic field. The RMS error rapidly decreases with increasing the number of disorder configurations.

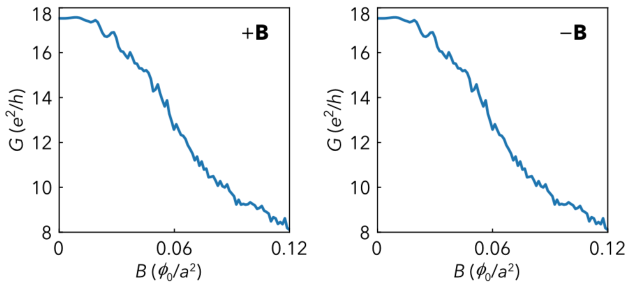

Supplementary Figure 14 | Comparison between the calculated magnetic fingerprints with opposite magnetic field directions. We checked whether the relationship required by the symmetry is satisfied. The same magnetic fingerprint appears with respect to the magnetic field reversal.

Supplementary Table 1 | Architecture of the convolution part of the feature extraction network.

Layer #	Layer name	Hyperparameters
1	Convolution	output channel=256, kernel size=4, stride=2, padding=1
2	Leaky ReLU	negative slope=0.2
3	Convolution	output channel=512, kernel size=4, stride=2, padding=1
4	Batch normalisation	
5	Leaky ReLU	negative slope=0.2
6	Linear	output features=1024
7	Batch normalisation	
8	Leaky ReLU	negative slope=0.2
9	Linear	output features=512
10-1	Linear (mean value)	output features=7
10-2	Linear (logarithm variance)	output features=7

Supplementary Table 2 | Architecture of the deconvolution part of the feature extraction network.

Layer #	Layer name	Hyperparameters
11	Linear	output features=2048
12	Batch normalisation	
13	ReLU	
14	Linear	output features=115200
15	Batch normalisation	
16	ReLU	
17	Transposed convolution	output channel=64, kernel size=4, stride=2, padding=1
18	Batch normalisation	
19	ReLU	
20	Transposed convolution	output channel=1, kernel size=4, stride=2, padding=1

Supplementary Table 3 | Architecture of the prediction part of the geometry generative network.

Layer #	Layer name	Hyperparameters
21	Linear	output features=8192
22	ReLU	
23	Dropout	Ratio=0.5
24	Linear	output features=8192
25	ReLU	
26	Dropout	Ratio=0.5
27	Linear	output features=8192
28	ReLU	
29	Dropout	Ratio=0.5
30	Linear	output features=7

Supplementary Note 1 | Experimental.

To apply QGD to data from real objects, fine tuning the network by using data from real objects is important, although it requires enormous experimental resources and a long time. In this section, to show the current state of QGD application, we simply show some experimental data.

On a trial basis, we apply the trained QGD without fine tuning to experimental data from a nano-structured Au film. In Supplementary Fig. 4a, we show a scanning electron microscope image for the sample. The sample was fabricated by the lift-off technique using electron beam lithography. A 25 nm-thick Au film with a width of 280 nm is deposited on a SiO_2/Si substrate in ultra-high vacuum. The Au wire has two damaged areas with diameters of ~50 nm. The damaged parts are made by irradiating an electron beam with an acceleration voltage of 50 kV and a beam current of 130 pA for 20 seconds. The magneto-resistance was measured by a four probe method with an a.c. resistance bridge (Lake Shore 372). The amplitude and frequency of the applied a.c. charge current were set to 3.16 μA and 11.6 Hz, respectively. The measurement was performed at 100 mK using a ^{3}He-^{4}He dilution refrigerator (KelvinoxMX200). The magnetic field was swept with a sweep rate of 12 mT/min. The field pattern of the resistance is almost same between data measured with decreasing and increasing the magnetic field applied perpendicular to the film.

The normalised magneto-conductance ΔG shown in Supplementary Fig. 4b is obtained as follows: first, the classical background of the conductance is subtracted using a 2nd order polynomial fitting. The conductance data is normalised by dividing the data by its standard deviation. Finally, the data is picked up at 0.025 T intervals to match the input size of the QGD. Supplementary Fig. 4c shows an output of the QGD from the conductance data.

Supplementary References

1. Kingma, D. P. & Welling, M. Auto-Encoding Variational Bayes. Preprint at https://arxiv.org/abs/1312.6114 (2013).
2. Doersch, C. Tutorial on Variational Autoencoders. Preprint at https://arxiv.org/abs/1606.05908 (2016).
3. Glorot, X., Bordes, A. & Bengio, Y. Deep Sparse Rectifier Neural Networks. In *Proc. 14th International Conference on Artificial Intelligence and Statistics* 315-323 (2011).
4. Xu, B., Wang, N., Chen, T. & Li, M. Empirical Evaluation of Rectified Activations in Convolutional Network. Preprint at https://arxiv.org/abs/1505.00853 (2015).
5. Ioffe, S. & Szegedy, C. Batch Normalization: Accelerating Deep Network Training by Reducing Internal Covariant Shift. Preprint at https://arxiv.org/abs/1502.03167 (2015).
6. Srivastava, N., Hinton, G., Krizhevsky, A., Sutskever, I. & Salakhutdinov, R. Dropout: A Simple Way to Prevent Neural Networks from Overfitting. *JMLR* **15,** 1929-1958 (2014).
7. Kingma, D. P. & Ba, J. Adam: A Method for Stochastic Optimization. Preprint at https://arxiv.org/abs/1412.6980 (2014).
